# Transcriptomic analyses of treatment-naïve pediatric ulcerative colitis patients and exploration of underlying disease pathogenesis

**DOI:** 10.1186/s12967-023-03881-6

**Published:** 2023-01-16

**Authors:** Xiaoli Pang, Hongxiao Song, Xiaolu Li, Fengchao Xu, Bingxun Lei, Fei Wang, Jing Xu, Lingli Qi, Libo Wang, Guangyun Tan

**Affiliations:** 1 Department of Pediatric Gastroenterology, The First Hospital of Jilin University, Changchun, China; 2 Department of Hepatology, Center for Pathogen Biology and Infectious Diseases, Institute of Translational Medicine, The First Hospital of Jilin University, Changchun, Jilin China; 3 Department of Anesthesia, The First Hospital of Jilin University, Changchun, China; 4 Health Examination Center, The First Hospital of Jilin University, Changchun, China

**Keywords:** Ulcerative colitis, Intestinal inflammation, Children, Intestinal fibrosis, Transcriptome analysis, HLA-DRB5, IL-1α

## Abstract

**Background:**

Ulcerative colitis (UC) is a form of chronic inflammatory bowel disease of nonspecific origin. This study used an RNA-Sequencing (RNA-Seq) approach to evaluate the transcriptomic landscape of a well-stratified treatment-naïve pediatric UC patient population by comparing them with healthy control children. The data were analyzed to evaluate the mechanisms driving UC-related intestinal inflammation and fibrosis.

**Methods:**

Intestinal mucosal samples from five pediatric UC patients and five healthy controls were analyzed by RNA-Seq, and results were verified by qPCR. A CRISPR/Cas9 approach was used to knock out the expression of HLA-DRB5, and molecular biology techniques were used for additional mechanistic studies.

**Results:**

In these analyses, 2290 genes were found to be differentially expressed between the UC and control samples, of which 1258 and 1032 were upregulated and downregulated, respectively. Gene Ontology analysis showed that these genes were enriched in extracellular matrix (ECM)-related processes and that 7 of 8 differentially expressed genes of interest (PIK3CD, IL1β, IL1α, TIMP1, MMP1, MMP12, COL6A3, and HLADRB5) were upregulated and involved in ECM-receptor interaction and inflammatory bowel disease-related pathways. Increased HLA-DRB5 expression driven by intestinal bacteria was found to promote IL-1α secretion, leading to intestinal inflammation and fibrosis, suggesting a possible target for the treatment of UC.

**Conclusion:**

These data suggest that intestinal inflammation is present in pediatric UC patients for extended periods before the onset of symptoms, and intestinal fibrosis begins even during the early stages of UC. Intestinal bacteria were also found to trigger intestinal inflammation and fibrosis, with HLA-DRB5 playing a central role in this process.

**Supplementary Information:**

The online version contains supplementary material available at 10.1186/s12967-023-03881-6.

## Background

Ulcerative colitis (UC) is a form of chronic intestinal inflammatory bowel disease (IBD) of poorly defined etiology. Adults with UC often experience abdominal pain and bloody diarrhea [1, 2]. In pediatric UC patients, the presentation of UC can differ from that in adults and is generally more severe, with rapid progression and a poorer prognostic outlook [3, 4]. Multicenter studies of European pediatric cohorts have suggested that pediatric-onset UC cases, which comprise 15–20% of all cases, affect 1–4 per 100,000 children per year in Europe and North America [5]. UC is extensive in 60–80% of all pediatric cases compared with just 20–30% of adult cases [6]. In children, UC is often likely to require hospitalization during acute episodes of severe disease, affecting 25–30% of patients over 3–4 years [7, 8]; these patients are more likely to undergo colectomy if the disease is refractory to treatment, which occurs in approximately 30–40% of patients undergoing such treatment over a 10-year follow-up interval [9]. Pediatric UC patients also exhibit other age-related clinical manifestations, including nutritional deficits, altered growth and pubertal development, bone mineral density accretion, and psychosocial needs, that are distinct from those of adults. There is thus a pressing need to further explore the specific etiology of pediatric UC to guide appropriate patient care.

Transcriptomic analyses can provide fundamental insights into the function of genes and regulatory structures, enabling detailed studies of disease pathogenesis. Most UC-related transcriptomic studies conducted to date have focused on adult patients [10–12]. These have shown that many of the disease-related dysregulated genes are associated with the maintenance of the epithelial barrier, including overexpression of genes associated with the mucus layer, consistent with the barrier dysfunction seen in these patients [13]. UC patients also show significantly increased expression of genes associated with innate pattern recognition receptors and their pathways, potentially highlighting targets that could guide intervention and treatment in affected patients [14].

There have been few transcriptomic studies of pediatric UC patients, although several studies on children with IBD have successfully identified biomarkers that could be used for differential diagnosis [15] and the prediction of clinical activity [16]. Gastrointestinal transcriptomic data from affected patients are even rarer, with one study observing the suppression of 13 mitochondrially encoded electron transport-related genes (Complex I, III, IV, and V), PPARGC1A (PGC1α), and epithelial MMP in both adult and pediatric UC mucosal samples [12]. By using purified intestinal epithelial cell samples for DNA methylation and transcriptomic analyses, Howell et al. demonstrated that these cells may change over the course of IBD development in a manner directly associated with disease pathogenesis [17]. The upregulation of genes associated with inflammation such as CEACAM1, MMP8, and ABCC4 (MRP4) in pediatric UC patients has also been linked to early resistance to corticosteroid treatment [18].

Here, transcriptomic analyses were performed using samples collected from a group of well-stratified newly-diagnosed pediatric UC patients to explore the underlying pathogenesis of intestinal inflammation and fibrosis. The results identified new potential regulators of the pathogenesis of treatment-naïve UC and support the occurrence of early intestinal fibrosis in these UC cases. Mechanistically, HLA-DRB5 was found to be an important regulator of intestinal inflammation and fibrosis. Together, these data may offer value for future studies of the pathogenic etiology of pediatric UC and the development of therapeutic strategies tailored to the treatment of these children.

## Material and methods

### Study participants

This study was conducted at the First Hospital of Jilin University (Changchun City, Jilin Province, China). Pediatric UC cases were diagnosed based on the results of endoscopic and histological analyses following the ECCO guidelines [19]. Mayo endoscopic scores were used to grade the severity of inflammation during colonoscopy, with scores of 1–2 corresponding to mild-to-moderate UC [20]. Children used as controls in this study were individuals who had undergone colonoscopy due to abdominal pain without fever or diarrhea and in whom the whole colonic histological, colonoscopy, abdominal imaging, and auxiliary examinations yielded normal results. Biopsy samples were collected from the rectum of all patients. The guardians of participants signed an informed and written consent form. The study was approved by Ethics Committee of the First Hospital of Jilin University [2019-349].

### RNA preparation

RNA was extracted using TRIzol, according to the provided directions. The purity of the RNA was assessed using agarose gel electrophoresis, which was also used to confirm the absence of DNA contamination. A nanophotometer was used to evaluate RNA purity (OD 260/280 and OD 260/230), and an Agilent 2100 Bioanalyzer was used for accurate quantification of RNA integrity.

### Library construction and sequencing

mRNA samples were randomly fragmented with divalent cations in NEB fragmentation buffer, after which libraries were prepared with a NEBNext^®^ Ultra^™^ RNA Library Prep Kit based on provided directions for Illumina^®^ sequencing (NEB, USA). An Agilent 2100 Bioanalyzer and an ABI StepOnePlus RT-PCR system were used for library assessment, and cDNA libraries of sufficient quality were added to flow cells and subjected to 125/150 bp paired-end sequencing with an Illumina HiSeq 4000 high-throughput sequencer.

### Sequencing data analyses

The raw data were filtered to remove poly-N-containing reads, adapter-containing reads, and low-quality reads. The resultant clean reads were then analyzed with HISAT2 and compared and annotated with the reference genome. Gene expression was analyzed in the Fragments Per Kilobases of transcript per Million mapped Reads (FPKM) format, and P-values were corrected for false discovery rate (FDR). Genes with an adjusted P < 0.05 were classified as differentially expressed genes (DEGs). The mainstream hierarchical clustering approach was used to cluster gene FPKM values, and rows were homogenized (Z-scores).

Gene Ontology (GO) and Kyoto Encyclopedia of Genes and Genomes (KEGG) enrichment analyses of the identified DEGs were conducted using the clusterProfiler package in R, with terms with adjusted P < 0.05 considered significantly enriched. Protein–protein interactions for the identified DEGs were analyzed with the STRING protein interaction database. Alternative splicing events were analyzed with rMATS, with a 5% FDR cut-off as the threshold for significant alternative splicing differences.

### qPCR

The extracted RNA was reverse-transcribed to cDNA that was then amplified by qPCR using the FastStart Universal SYBR Green Master (ROX) (Roche, Germany). The ΔΔCT method was used to assess relative gene expression, and target gene expression was normalized to GAPDH mRNA levels.

### Western immunoblotting

Ice-cold lysis buffer was used to lyse cells, and equal amounts of protein from each sample were separated on 12.5% SDS-PAGE and transferred to nitrocellulose membranes. The blots were blocked using 5% non-fat milk in PBST (PBS containing 0.05% Tween 20) followed by overnight incubation with the primary antibodies anti-HLA-DRB5 or anti- IL-1α at 4 °C. After washing with PBST, the blots were incubated at room temperature for 2 h with appropriate secondary antibodies, washed three additional times, and proteins were detected by enhanced chemiluminescence.

### CRISPR/Cas9 knockout

Caco2 cells were seeded in 24-well plates. After 16 h, two plasmids, one expressing Cas9 with HLA-DRB5 sgRNA (FG-EH-Cas9-2F-PPW) and the other carrying a puromycin-resistant gene (PL-GFP-IP), were co-transfected into Caco2 cells using the ViaFect transfection reagent (Promega). Thirty-six hours after transfection, the cells were either selected by adding puromycin at a concentration of 2 μg/ml or collected for immunoblotting with specific HLA-DRB5 antibodies. The SgRNAs are listed in Additional file 1: Table S1.

### Statistical analysis

Data were analyzed using GraphPad Prism 5 and compared using t-tests or ANOVA. P < 0.05 was the threshold of significance; *P < 0.05, ** P < 0.01, *** P < 0.001.

## Results

### Patient characteristics

For this study, five pediatric UC patients and five age- and sex-matched controls were recruited for RNA-Seq analyses. The control children were free of fevers and diarrhea, exhibited normal white blood cell (WBC) counts, C-reactive protein (CRP) levels, and erythrocyte values, and had normal colonic histology, colonoscopy, and abdominal imaging results. The UC patients showed bloody stool, elevated WBC, CRP, and/or ESR levels, and evidence of intestinal ulceration and inflammation observed by colonoscopy and had been diagnosed in accordance with the ECCO guidelines [10]. The Pediatric Ulcerative Colitis Activity Index (PUCAI) values for these patients ranged from 20 to 60, consistent with mild-to-moderate disease. None of the patients had undergone specific treatment before sample collection.

### Transcriptomic analyses

**Fig. 1:**
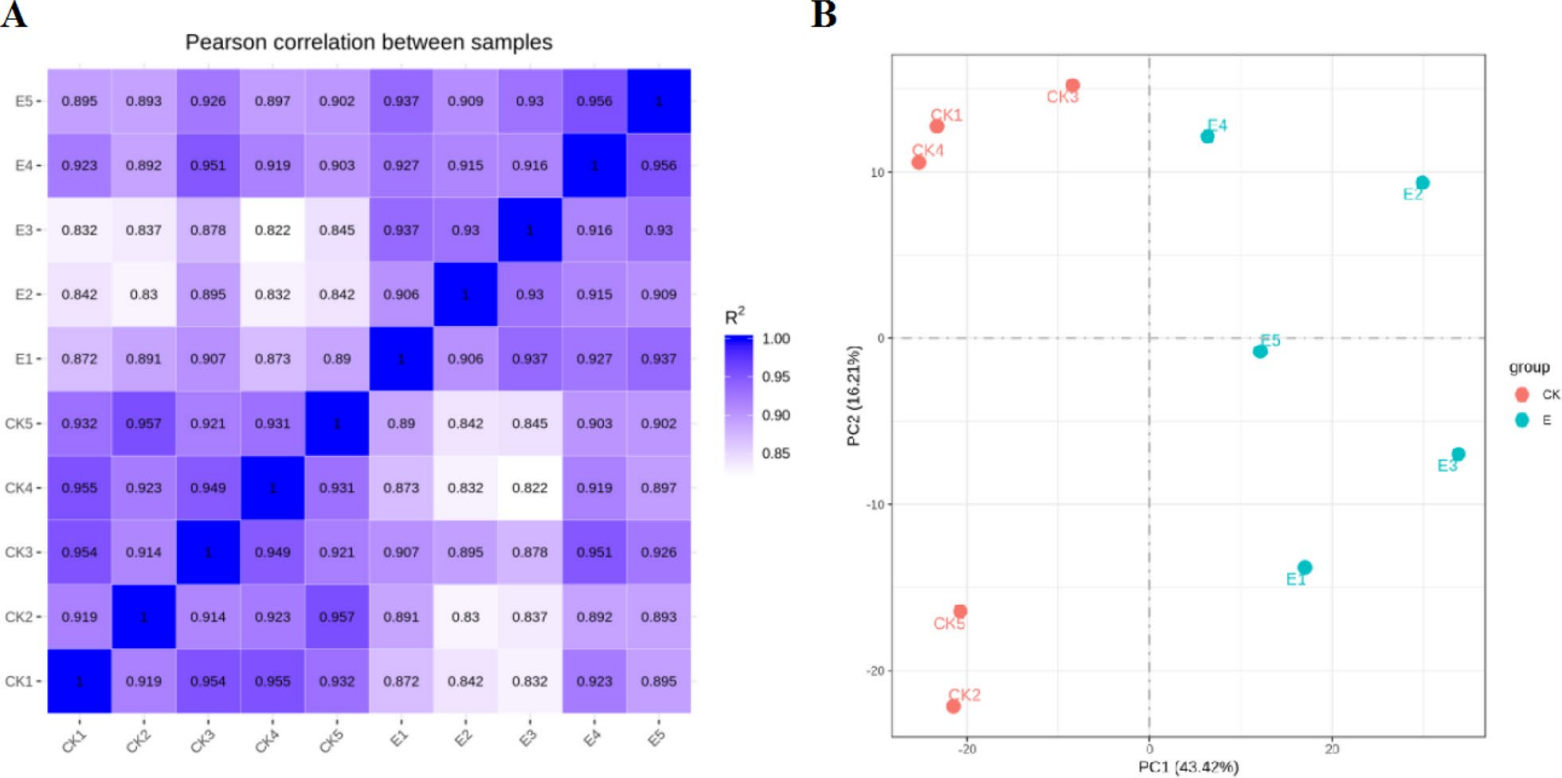
Analyses of the transcriptomic profiles of intestinal mucosal samples from pediatric UC and control patients. **A** A correlation coefficient heatmap for the UC and control groups, revealing highly consistent measurements within groups, *R*^2^ ≥ 0.822. **B** A PCA plot was used to cluster samples, revealing good repeatability. Each point represents one sample, green and red circles respectively correspond to UC and control samples, and percentages denote contribution ratios

To explore UC-related changes in the transcriptomic landscape of the intestinal mucosa, total RNA was isolated from mucosal samples from the 10 patients and sequenced. After the removal of low-quality and ambiguous reads, each sample yielded 44–57 million clean paired reads (Additional file 2: Table S2). Over 85% of these read pairs were successfully aligned with the human reference genome. Standardized transcript levels were analyzed using FPKM values, and correlation matrices showed that the results were highly consistent within the two patient groups (R^2^ ≥ 0.822; Fig. 1A). Samples were clustered using principal component analysis (PCA), showing clear separation of the clusters of the two groups (Fig. 1B).

### DEG identification and GO annotation

**Fig. 2:**
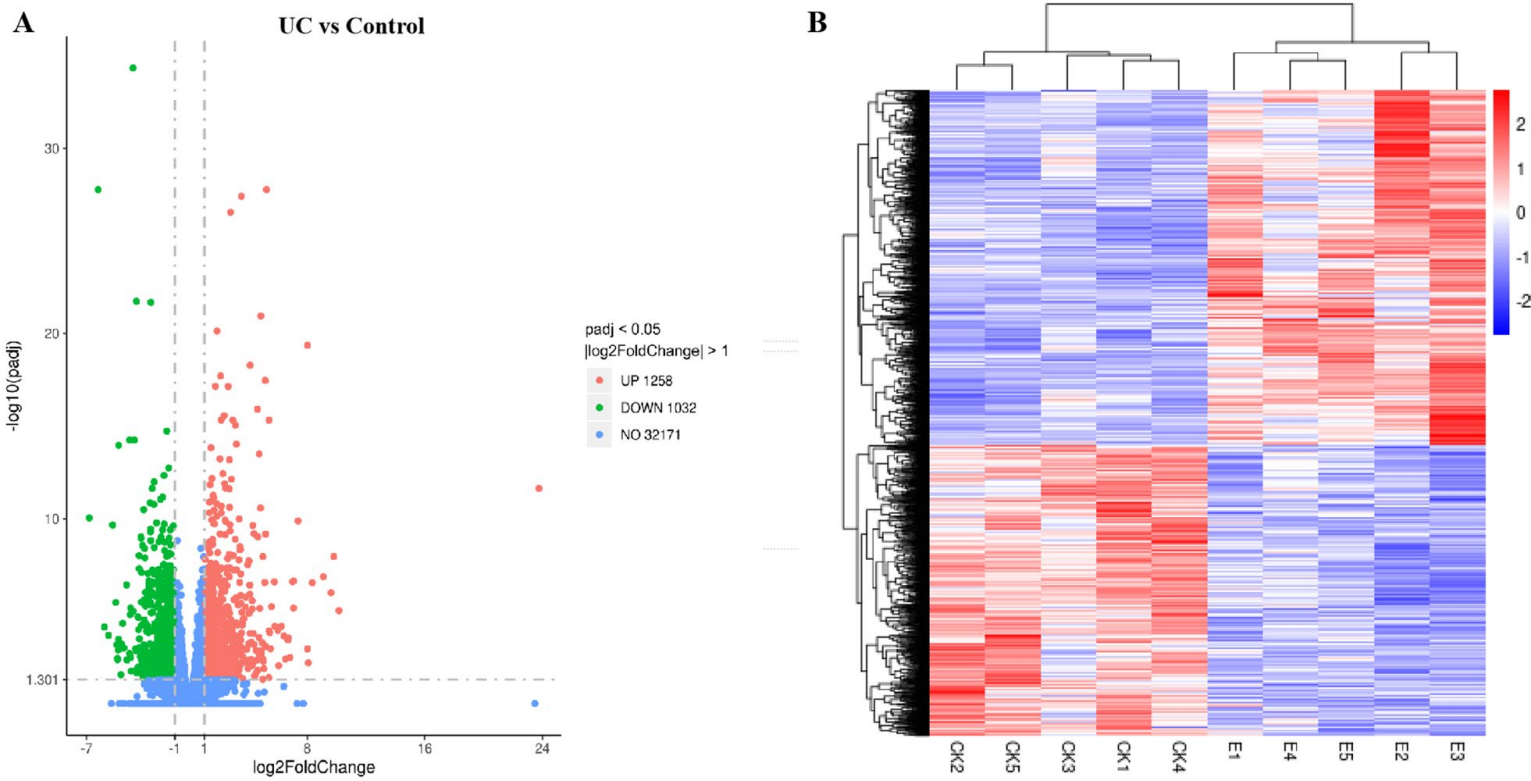
Identification of UC-associated DEGs in the intestinal mucosa. **A** A volcano plot of DEGs identified when comparing the UC and control groups, with each point corresponding to an individual gene. Red and green points denote upregulated and downregulated genes, respectively, while non-significant genes are shown in blue. **B** A clustering analysis of all significantly (adjusted P < 0.05) DEGs identified when comparing the UC and control group samples (CK represents control group and E represents UC group)

In total, 2,290 DEGs were identified when comparing the UC and control groups, of which 1,258 and 1,032 were up- and downregulated, respectively (Fig. 2A and Additional file 3: Table S3). Clustering analyses of the DEGs are shown in Fig. 2B. The 20 genes that were most highly upregulated in UC were SPINK4, CCL11, LRP8, SLC6A20, VWF, MMP10, SERPINB5, ARNTL2, MMP3, LIPG, PDPN, AQP9, SELP, TNC, LOXL2, VSIG1, ADAMTS12, STRIP2, LPIN1, and TREM1.

**Fig. 3:**
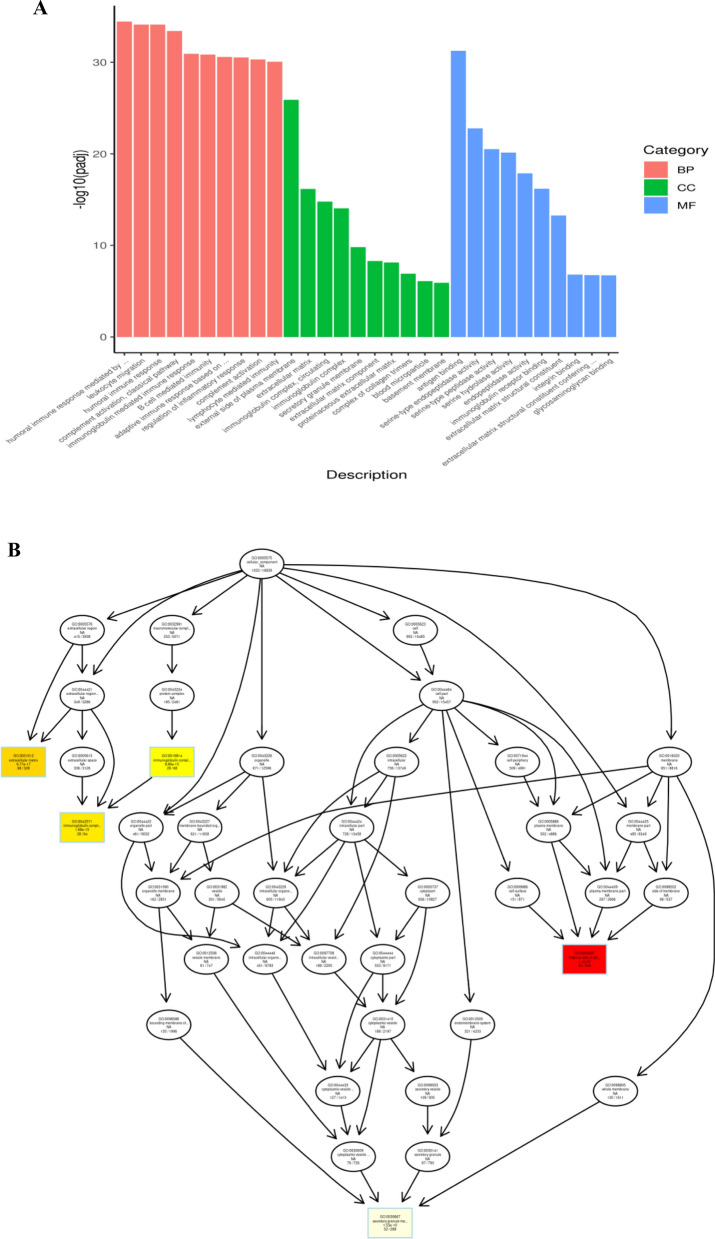
GO analyses of upexpressed genes between UC and control samples. **A** Significant GO terms were enriched in the biological process (BP), cellular component (CC) and molecular function (MF) categories. The numbers of upregulated genes between UC and control samples in each category were compared. **B** A directed acyclic graph of GO enrichment for upregulated genes, with squares indicating the top 5 GO terms based on adjusted p-values and the intensity of the red color indicating the degree of enrichment

To begin examining the biological roles of the DEGs, GO annotation analyses were conducted to assess their enrichment in specific biological process (BP), cellular component (CC) and molecular function (MF), classifications, as shown in Fig. 3A where the number of mRNAs associated with each term is shown along the horizontal axis. In total, 437 significant GO terms were identified (P.adj ≤ 0.01), and the 30 most significantly GO distributions of upregulated genes between the UC and control samples are listed in Additional file 4: Table S4. A directed acyclic graph was used to represent the relationships among identified enriched CC terms (Fig. 3B). Overall, the identified DEGs were enriched in several extracellular matrix (ECM)-related GO terms including extracellular structure organization (BP GO:0043062), extracellular matrix organization (BP GO:0030198), extracellular matrix (CC GO:0031012), extracellular matrix component (CC GO:0044420), complex of collagen trimers (CC GO:0098644), and extracellular matrix structural constituent (MF GO:0005201).

### KEGG pathway enrichment analysis

Next, DEGs were subjected to a KEGG pathway enrichment analysis that identified 296 enriched pathways. 25 significantly enriched pathways associated with 530 up-regulated genes (P.adj ≤ 0.05) were identified (Additional file 5: Table S5), including the Cytokine-cytokine receptor interaction, ECM-receptor interaction, PI3K-Akt signaling pathway, Inflammatory bowel disease (IBD), etc.

Based on a combination of GO results, KEGG pathways, and genes of interest, 8 DEGs were identified including PIK3CD, IL1β, IL1α, TIMP1, MMP1, MMP12, COL6A3, and HLADRB5, of which 7 were associated with intestinal inflammation and fibrosis in UC, and involved in KEGG pathways associated with the ECM-receptor interaction and inflammatory bowel disease (Additional file 6: Table S6). This finding suggests the coexistence of intestinal inflammation and fibrosis even during the early stages of pediatric UC.

**Fig. 4:**
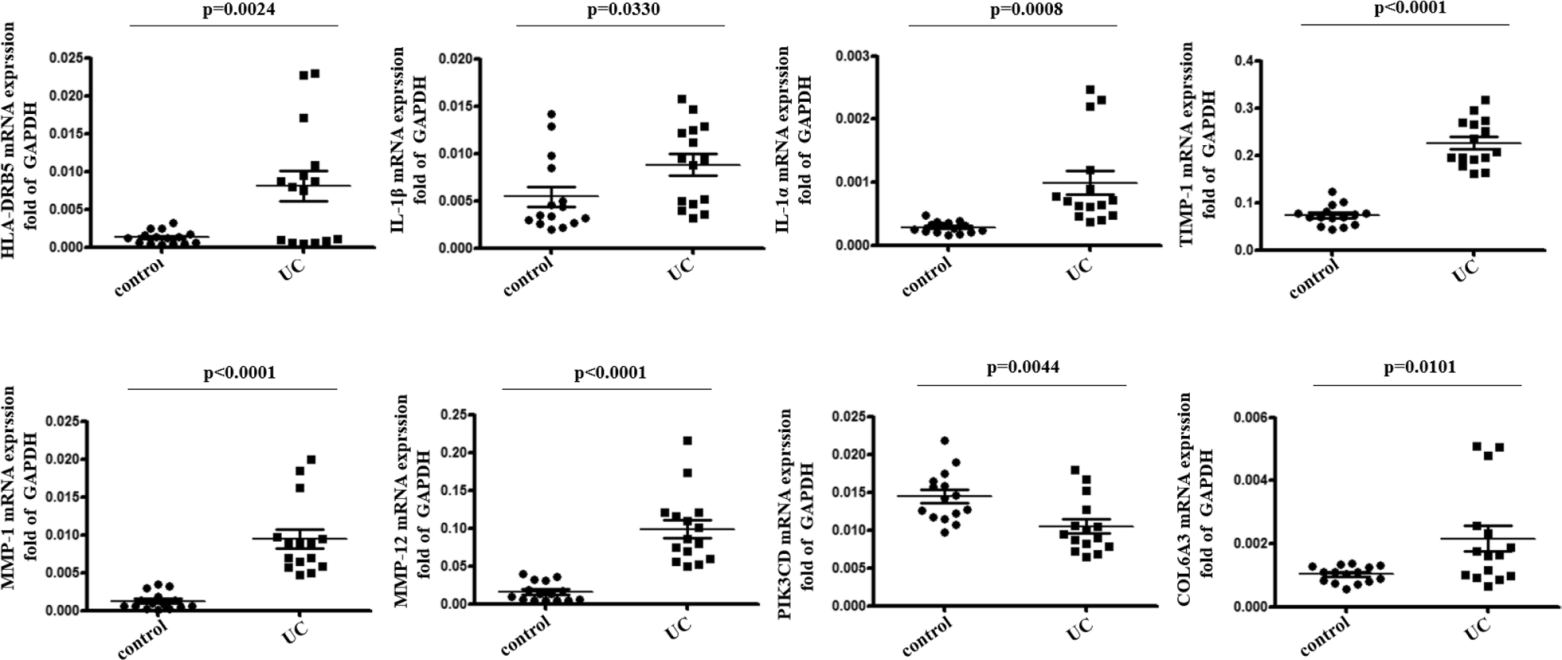
Verification of DEGs identified when comparing UC and control samples. qPCR verification of DEGs of interest

To confirm these findings, qPCR was used to assess the expression of these 8 DEGs in intestinal mucosal tissue samples from children with UC and controls (Additional file 7: Table S7) using the primers (Biotree, Shanghai, China) listed in Additional file 8: Table S8. All of these genes were significantly differentially expressed in UC patient samples relative to controls (Fig. 4), confirming the above transcriptomic results. However, while PIK3CD was upregulated in UC samples relative to controls in the RNA-Seq analyses, it was found to be downregulated on qPCR verification (Fig. 4). Nevertheless, both up- and downregulation are indicative of altered immune functionality [21–23].

### Intestinal inflammation and fibrosis in pediatric UC

The intestines contain high levels of bacteria, including many Gram-negative species that have cell walls composed primarily of lipopolysaccharide (LPS), which can stimulate an innate immune response in host cells and often plays a major role in the pathogenesis of many bacterial infections.

**Fig. 5:**
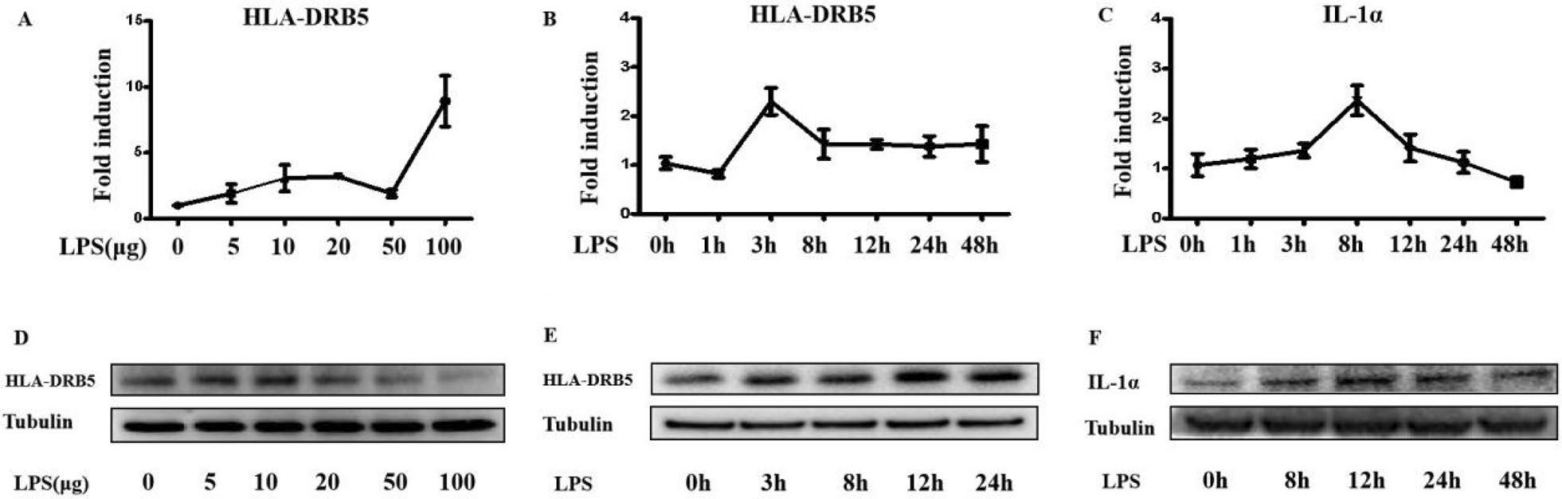
LPS stimulation promotes HLA-DRB5 and IL-1α upregulation. **A**, **D **Caco2 cells were treated with a range of LPS concentrations for 3 h, after which cells were collected and HLA-DRB5 expression was analyzed via qPCR and Western immunoblotting. **B, C, E, F** Caco2 cells were treated with 10 μg/ml of LPS and cultured for the indicated amounts of time, after which HLA-DRB5 and IL-1α expression levels were assessed via qPCR and Western immunoblotting

To simulate the effect of intestinal bacteria, Caco2 intestinal epithelial cells were cultured in the presence of LPS (10 μg/ml), resulting in HLA-DRB5 upregulation at the mRNA level. Peak HLA-DRB5 expression was observed at 3 h post-LPS treatment, whereas IL-1α expression peaked at 8 h post-LPS treatment. The highest HLA-DRB5 and IL-1α protein levels in these cells were detected at 12 h following stimulation (Fig. 5A–F).

**Fig. 6:**
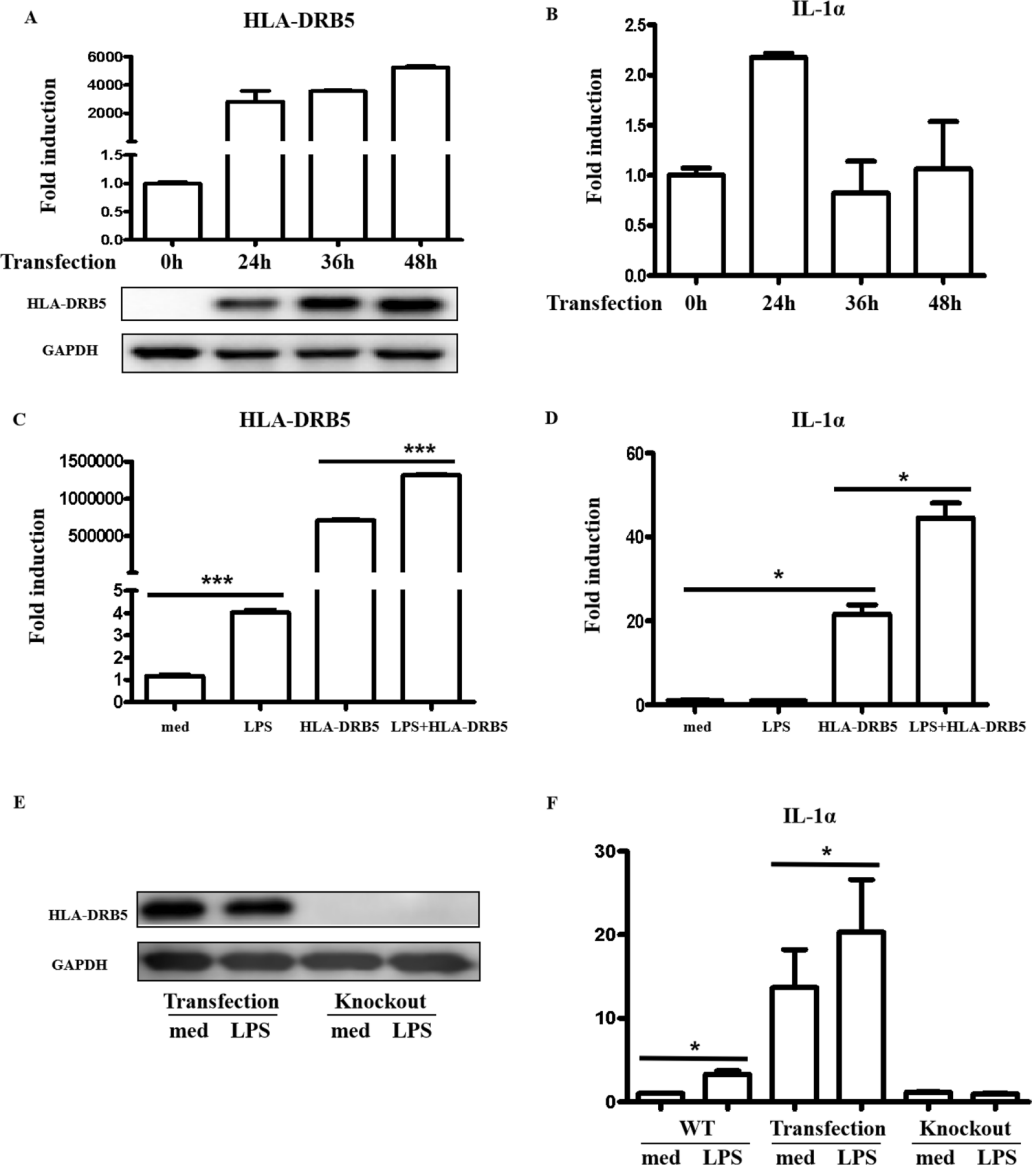
Knockout of HLA-DRB5 in Caco2 cells inhibits LPS-induced IL-1α expression. **A**, **B** An HLA-DRB5 expression plasmid was introduced into Caco2 cells, and the expression of this gene and IL-1α was assessed over time using qPCR and Western immunoblotting. **C**, **D** Caco2 cells were analyzed in control, LPS treatment (10 μg/ml), HLA-DRB5, and HLA-DRB5 + LPS groups. Cells were collected at 3 h post-LPS treatment and 24 h post-HLA-DRB5 transfection to assess HLA-DRB5 and IL-1α expression by qPCR. **E**, **F** Control, HLA-DRB5 overexpression, and HLA-DRB5 knockout groups were analyzed, with cells collected 8 h post-LPS stimulation and 24 h post-HLA-DRB5 transfection to measure IL-1α expression by qPCR

An HLA-DRB5 expression construct was next prepared and transfected into Caco2 cells, resulting in a significant rise in HLA-DRB5 after 24 h (Fig. 6A). IL-1α levels also rose significantly at 24 h after HLA-DRB5 overexpression (Fig. 6B). HLA-DRB5 and IL-1α expression levels increased significantly following LPS treatment after HLA-DRB5 overexpression (Fig. 6C, D), suggesting that HLA-DRB5 serves as a key mediator of IL-1α induction.

Next, HLA-DRB5 was knocked out by generating an sgRNA construct targeting the exonic sequence of this gene as recorded in the NCBI database using appropriate CRISPR software (http://crisper.mit.edu/). Plasmids expressing sgRNA and Cas9 were then transfected into Caco2 cells to knock out HLA-DRB5, which was confirmed by Western immunoblotting (Fig. 6E). While LPS increased IL-1α expression in control cells after 8 h, there was no change in its expression after HLA-DRB5 knockout (Fig. 6F). These results strongly suggested that HLA-DRB5 upregulation in response to LPS is required for IL-1α induction.

**Fig. 7:**
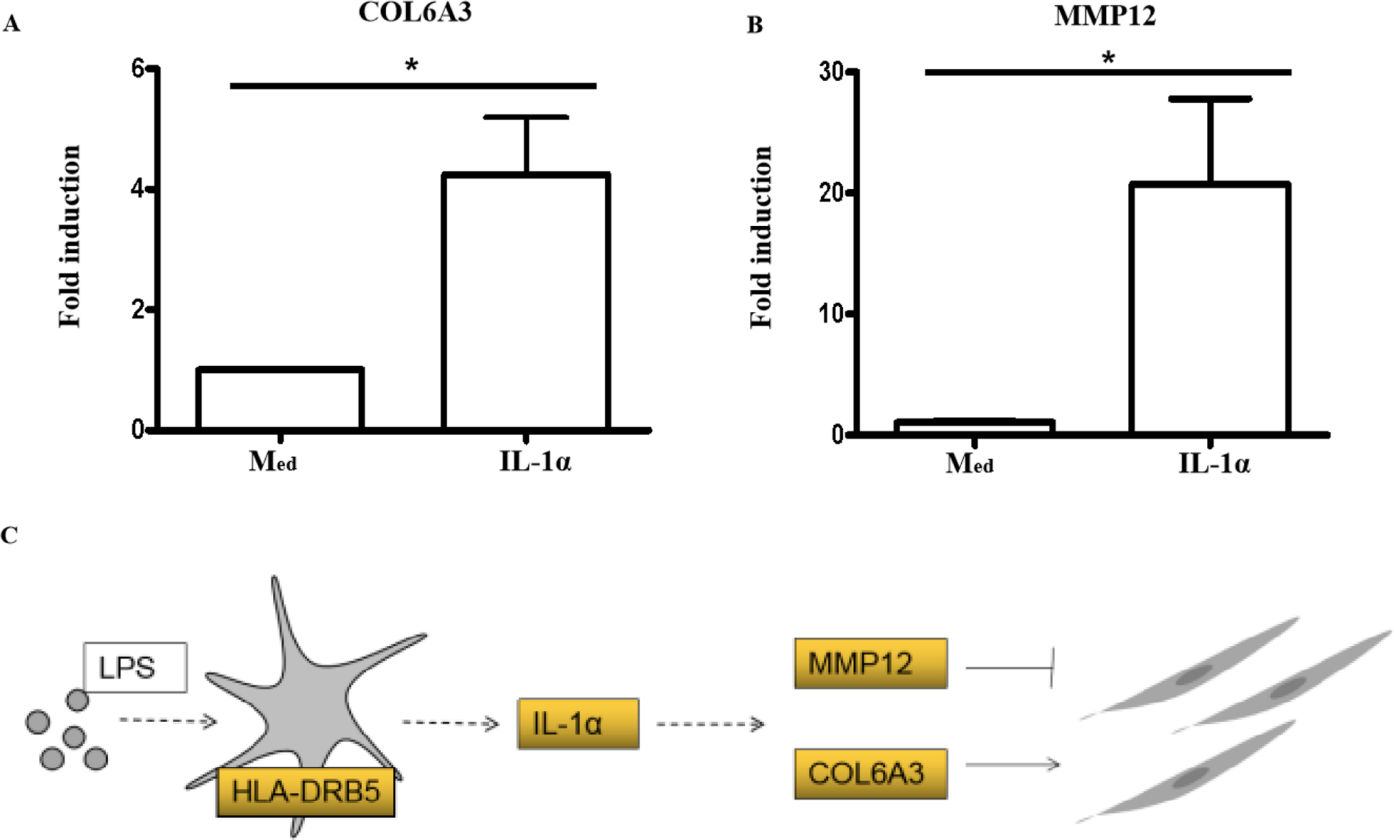
IL-1α promotes intestinal inflammation and fibrotic activity in pediatric UC. **A**, **B** Caco2 cells were treated with IL-1α (20 ng/ml) for 8 h, and then RNA was collected to assess COL6A3 and MMP12 expression by qPCR. **C** A diagram representing the mechanisms driving intestinal inflammation and fibrosis in pediatric UC

LPS induced HLA-DRB5 expression and HLA-DRB5, in turn, promoted IL-1α production. In addition, IL-1α was able to promote the intestinal epithelial-mesenchymal transition, contributing to higher levels of COL6A3 and MMP12 expression (Fig. 7A, B).

Together these data suggest that in response to intestinal bacteria, intestinal epithelial cells upregulate HLA-DRB5, thereby promoting IL-1α secretion. This cytokine, in turn, promotes intestinal inflammation, the epithelial-mesenchymal transition, and COL6A3 and MMP12 expression. COL6A3 can support microfibril formation and ECM deposition, contributing to intestinal fibrosis, whereas MMP12 can break down components of the ECM to protect against fibrosis. When the balance between these mechanisms is disrupted, intestinal fibrosis can occur (Fig. 7C).

Other pro- and anti-fibrotic factors including TIMP-1 and MMP1 may also play roles in this context. While LPS stimulation and HLA-DRB5 transfection had no effect on the expression of IL-1β in intestinal epithelial cells (data not shown), IL-1β is a key cytokine necessary for intestinal inflammatory response induction, and other pathways may thus govern IL-1β expression and secretion in this context.

## Discussion

UC is a form of chronic nonspecific IBD for which there is no available cure, and it appears to be becoming increasingly common in children. Many studies have explored the pathogenesis of UC in adults [24–26], whereas comparable studies in children are rare. Children with UC are more likely to experience relapse, exhibit a wide range of lesions, generally have a longer disease course, and are more likely to experience complications and need to undergo surgery. This study was thus designed to better understand the etiology of pediatric UC and define factors that may be used in the treatment of this condition.

In this transcriptomic study of pediatric UC patient samples, 1,258 and 1,032 genes were found to be upregulated and downregulated, respectively, relative to controls. These genes were enriched in a range of ECM-related GO terms, and some were also enriched in the ECM-receptor interaction pathways identified by KEGG, suggesting that intestinal fibrosis may be present even during early-stage UC.

Previous studies have suggested the importance of class II HLA genes in the regulation of UC pathogenesis. Indeed, high levels of HLA-DR expression have been detected in intestinal epithelial samples from UC patients [27], and HLA-DR expression has also been closely linked to mucosal inflammation [28, 29]. The HLA-DRB1*103 [30] and HLA-DRB1*12 alleles are specifically associated with the pathogenesis of UC [31], serving as genetic determinants of patient susceptibility that can also influence the presentation of this disease [32]. The present data are consistent with these findings, as upregulation of HLA-DRB5 was observed in intestinal epithelial cells in association with increased secretion of the inflammatory cytokine IL-1α. The release of IL-1α from damaged intestinal epithelial cells can contribute to chronic intestinal inflammation [33]. The present data also suggest that IL-1α may contribute to the development of intestinal fibrosis. The secretion of anti-fibrotic factors can also influence ECM degradation. Overall, our results suggested that intestinal bacteria initially trigger inflammation in the intestine and that this inflammation, in turn, contributes to the development of intestinal fibrosis. By the time these pediatric UC patients present to the clinic for disease-related symptoms, they may already have experienced intestinal inflammation at some level for some time due to these mechanisms. Importantly, the present data suggest that both inflammation and fibrosis occur in the intestines of pediatric UC patients during the early stages of disease. Normally, pro- and anti-fibrotic factors regulate the synthesis and degradation of the ECM. When this homeostatic balance is disrupted, however, intestinal fibrosis can develop.

Importantly, this study only included patient samples from treatment-naïve pediatric UC patients, providing a unique opportunity to understand UC-related transcriptomic profiles not affected by any medications. The treatment of UC patients with immunosuppressants and other drugs has been reported to alter immune function [34], potentially biasing experimental efforts seeking to understand the molecular etiology of UC in the absence of medical treatment.

There are some limitations to this study. For one, this was a transcriptomic analysis of samples from just 10 children, and additional large-scale analyses will be critical for further verification. Nevertheless, the samples included in the study were carefully selected and matched between the UC and control groups. Moreover, quality control and correlation matrix analyses revealed a high degree of consistency between the measurement results within each group and the observed changes in gene expression were confirmed by qPCR, reinforcing these conclusions.

## Conclusions

In summary, these data provide novel evidence through the RNA-sequencing of well-stratified intestinal mucosal samples from treatment-naïve pediatric UC patients that intestinal bacteria may trigger intestinal inflammation and subsequent intestinal fibrosis in children affected by this disease. Moreover, HLA-DRB5 was identified as a key mediator of these inflammatory and fibrotic responses, highlighting important targets for future UC treatment. Future studies will investigate the development of potential drugs targeting these factors.

### Limitations and strengths of this study

The limitation of this study: the sample size was small, with only 10 samples.

The strengths of this study:

1. The study only included patient samples from treatment-naïve pediatric UC patients, thus avoiding the effects of medication on the findings.
2. This is the first exploration of the problem of intestinal fibrosis in the early UC stage, reminding clinicians and researchers to pay more attention to intestinal fibrosis.
3. HLA-DRB5 was found to play a key regulatory role in intestinal inflammation and fibrosis, suggesting a potential therapeutic target for UC treatment.

## Supplementary Information

**Additional file 1: Table S1. **Primers sequences for quantitative real-time PCR and sgRNA.

**Additional file 2: Table S2.** Summary of the RNA sequencing reads and their mapping results.

**Additional file 3: Table S3.** Top 400 up-regulated genes of UC group vs control group.

**Additional file 4: Table S4.** Top 30 gene ontology distributions of up-expressed genes between UC group and control group.

**Additional file 5: Table S5.** KEGG pathway enrichment of upregulated mRNA in UC group and control group.

**Additional file 6: Table S6.** KEGG pathway enrichment.

**Additional file 7: Table S7.** Clinical characteristics of the patients involved in sequencing.

**Additional file 8: Table S8.** Primers used in qRT-PCR.

## Data Availability

The datasets supporting the conclusions of this article are included within the article and its additional files.

## Abbreviations

UC: Ulcerative colitis
IBD: Inflammatory bowel disease
ECM: Extracellular matrix
FPKM: Fragments per kilobases of transcript per million mapped reads
LPS: Lipopolysaccharide
FDR: False discovery rate
GO: Gene ontology
KEGG: Kyoto Encyclopedia of Genes and Genomes
qRT-PCR: Quantitative real-time polymerase chain reaction
cDNA: Complementary DNA
ΔΔCT: Comparative CT
WBC: White blood cell
ESR: Erythrocyte sedimentation rate
CRP: C reaction protein
PUCAI: Pediatric Ulcerative Colitis Activity Index
PCA: Principal component analysis
