# Supplementary material for: Transcriptomic analyses of treatment-naïve pediatric ulcerative colitis patients and exploration of underlying disease pathogenesis
Source: J Transl Med. 2023 Jan 16;21:30. doi: 10.1186/s12967-023-03881-6 (PMC9843999; doi:10.1186/s12967-023-03881-6)
Supplement: Supplementary file 1 — Additional file 1: Table S1. Primers sequences for quantitative real-time PCR and sgRNA. [file 12967_2023_3881_MOESM1_ESM.doc]

**Table S1. Primers sequences for quantitative real-time PCR and sgRNA**

| Primers | | Sequence |
| --- | --- | --- |
| GAPDH | Forward | 5'-CGGATTTGGTCGTATTGGG-3' |
| GAPDH | Reverse | 5'-TCTCGCTCCTGGAAGATGG-3' |
| HLADR5 sgRNA | Forward | CACC GAACAGCCAGAAGGACTTCC |
| HLADR5 sgRNA | Reverse | AAAC GGAAGTCCTTCTGGCTGTTC |
