# Supplementary material for: Transcriptomic analyses of treatment-naïve pediatric ulcerative colitis patients and exploration of underlying disease pathogenesis
Source: J Transl Med. 2023 Jan 16;21:30. doi: 10.1186/s12967-023-03881-6 (PMC9843999; doi:10.1186/s12967-023-03881-6)
Supplement: Supplementary file 2 — Additional file 2: Table S2. Summary of the RNA sequencing reads and their mapping results. [file 12967_2023_3881_MOESM2_ESM.doc]

**Table S2**. Summary of the RNA sequencing reads and their mapping results.

| Sample name | UC1 | UC 2 | UC 3 | UC 4 | UC 5 | Control 1 | Control 2 | Control 3 | Control 4 | Control 5 |
| --- | --- | --- | --- | --- | --- | --- | --- | --- | --- | --- |
| Total reads | 51699100 | 54992450 | 48557276 | 48490678 | 46259486 | 56639028 | 50717440 | 57695570 | 52034298 | 44944486 |
| Total map | 49079714  (94.93%) | 51886515  (94.35%) | 45086664  (92.85%) | 46020087  (94.91%) | 43576261  (94.2%) | 53998302  (95.34%) | 48118676  (94.88%) | 54639454  (94.7%) | 49498718  (95.13%) | 42739051  (95.09%) |
| Unique map | 47458230  (91.8%) | 49977832  (90.88%) | 43189724  (88.95%) | 44662710  (92.11%) | 42282551  (91.4%) | 52405870  (92.53%) | 46761744  (92.2%) | 52844228  (91.59%) | 47944522  (92.14%) | 41498423  (92.33%) |
| Multiple map | 1621484  (3.14%) | 1908683  (3.47%) | 1896940  (3.91%) | 1357377  (2.8%) | 1293710  (2.8%) | 1592432  (2.81%) | 1356932  (2.68%) | 1795226  (3.11%) | 1554196  (2.99%) | 1240628  (2.76%) |
| Positive map | 23726845  (45.89%) | 24974126  (45.41%) | 21602057  (44.49%) | 22322575  (46.03%) | 21126991  (45.67%) | 26203437  (46.26%) | 23356121  (46.05%) | 26413222  (45.78%) | 23957709  (46.04%) | 20733558  (46.13%) |
| Negative map | 23731385  (45.9%) | 25003706  (45.47%) | 21587667  (44.46%) | 22340135  (46.07%) | 21155560  (45.73%) | 26202433  (46.26%) | 23405623  (46.15%) | 26431006  (45.81%) | 23986813  (46.1%) | 20764865  (46.2%) |
| Splice map | 18244936  (35.29%) | 21315557  (38.76%) | 17663606  (36.38%) | 18632133  (38.42%) | 17397421  (37.61%) | 18009818  (31.8%) | 16392440  (32.32%) | 20723657  (35.92%) | 17983704  (34.56%) | 15574915  (34.65%) |
| Unsplice map | 29213294  (56.51%) | 28662275  (52.12%) | 25526118  (52.57%) | 26030577  (53.68%) | 24885130  (53.79%) | 34396052  (60.73%) | 30369304  (59.88%) | 32120571  (55.67%) | 29960818  (57.58%) | 25923508  (57.68%) |

Total reads: The number of clean reads after sequencing data filtering.

Total map: The number and percentage of reads which can map to the genome.

Unique map: The number and percentage of reads which have Single location on the reference sequencing.

Multiple map: The number and percentage of reads which have multiple location on the reference sequencing.

Positive map: The number and percentage of reads which were mapped on the ‘+’chain of the genome.

Negative map: The number and percentage of reads which were mapped on the ‘-’chain of the genome.

Splice map：The number and percentage of reads which were spliced and mapped on two exons of the genome.

Unsplice_map：The number and percentage of reads which completely were mapped on two exons of the genome
