# Supplementary material for: Transcriptomic analyses of treatment-naïve pediatric ulcerative colitis patients and exploration of underlying disease pathogenesis
Source: J Transl Med. 2023 Jan 16;21:30. doi: 10.1186/s12967-023-03881-6 (PMC9843999; doi:10.1186/s12967-023-03881-6)
Supplement: Supplementary file 3 — Additional file 3: Table S3. Top 400 up-regulated genes of UC group vs control group. [file 12967_2023_3881_MOESM3_ESM.doc]

| Gene_ID  **Table S3. Top 400 up-regulated genes of UC group vs control group.** | Gene Name | log2FoldChange | padj | Gene_Description |
| --- | --- | --- | --- | --- |
| ENSG00000122711 | SPINK4 | 5.225521266 | 1.72E-28 | serine peptidase inhibitor, Kazal type 4 [Source:HGNC Symbol;Acc:HGNC:16646] |
| ENSG00000172156 | CCL11 | 3.525046895 | 3.96E-28 | C-C motif chemokine ligand 11 [Source:HGNC Symbol;Acc:HGNC:10610] |
| ENSG00000157193 | LRP8 | 2.789018377 | 2.86E-27 | LDL receptor related protein 8 [Source:HGNC Symbol;Acc:HGNC:6700] |
| ENSG00000163817 | SLC6A20 | 4.838809421 | 1.16E-21 | solute carrier family 6 member 20 [Source:HGNC Symbol;Acc:HGNC:30927] |
| ENSG00000110799 | VWF | 1.857767061 | 7.41E-21 | von Willebrand factor [Source:HGNC Symbol;Acc:HGNC:12726] |
| ENSG00000166670 | MMP10 | 8.017625611 | 4.44E-20 | matrix metallopeptidase 10 [Source:HGNC Symbol;Acc:HGNC:7156] |
| ENSG00000206075 | SERPINB5 | 4.117320884 | 5.40E-19 | serpin family B member 5 [Source:HGNC Symbol;Acc:HGNC:8949] |
| ENSG00000029153 | ARNTL2 | 2.11059946 | 2.02E-18 | aryl hydrocarbon receptor nuclear translocator like 2 [Source:HGNC Symbol;Acc:HGNC:18984] |
| ENSG00000149968 | MMP3 | 5.138122758 | 3.58E-18 | matrix metallopeptidase 3 [Source:HGNC Symbol;Acc:HGNC:7173] |
| ENSG00000101670 | LIPG | 1.75855764 | 7.31E-18 | lipase G, endothelial type [Source:HGNC Symbol;Acc:HGNC:6623] |
| ENSG00000162493 | PDPN | 2.621962564 | 7.45E-18 | podoplanin [Source:HGNC Symbol;Acc:HGNC:29602] |
| ENSG00000103569 | AQP9 | 4.617707965 | 1.20E-16 | aquaporin 9 [Source:HGNC Symbol;Acc:HGNC:643] |
| ENSG00000174175 | SELP | 2.351247344 | 2.69E-16 | selectin P [Source:HGNC Symbol;Acc:HGNC:10721] |
| ENSG00000041982 | TNC | 2.92590305 | 4.66E-16 | tenascin C [Source:HGNC Symbol;Acc:HGNC:5318] |
| ENSG00000134013 | LOXL2 | 2.156108904 | 4.66E-16 | lysyl oxidase like 2 [Source:HGNC Symbol;Acc:HGNC:6666] |
| ENSG00000101842 | VSIG1 | 5.406255495 | 4.70E-16 | V-set and immunoglobulin domain containing 1 [Source:HGNC Symbol;Acc:HGNC:28675] |
| ENSG00000151388 | ADAMTS12 | 3.120719002 | 8.89E-16 | ADAM metallopeptidase with thrombospondin type 1 motif 12 [Source:HGNC Symbol;Acc:HGNC:14605] |
| ENSG00000128578 | STRIP2 | 3.183930489 | 9.39E-15 | striatin interacting protein 2 [Source:HGNC Symbol;Acc:HGNC:22209] |
| ENSG00000134324 | LPIN1 | 1.455079279 | 1.44E-14 | lipin 1 [Source:HGNC Symbol;Acc:HGNC:13345] |
| ENSG00000124731 | TREM1 | 4.737985479 | 3.19E-14 | triggering receptor expressed on myeloid cells 1 [Source:HGNC Symbol;Acc:HGNC:17760] |
| ENSG00000197930 | ERO1A | 2.066489424 | 5.85E-14 | endoplasmic reticulum oxidoreductase 1 alpha [Source:HGNC Symbol;Acc:HGNC:13280] |
| ENSG00000187498 | COL4A1 | 2.707278878 | 6.65E-14 | collagen type IV alpha 1 chain [Source:HGNC Symbol;Acc:HGNC:2202] |
| ENSG00000149485 | FADS1 | 2.261243034 | 3.69E-13 | fatty acid desaturase 1 [Source:HGNC Symbol;Acc:HGNC:3574] |
| ENSG00000118193 | KIF14 | 1.521756232 | 6.67E-13 | kinesin family member 14 [Source:HGNC Symbol;Acc:HGNC:19181] |
| ENSG00000196352 | CD55 | 2.852853157 | 7.32E-13 | CD55 molecule (Cromer blood group) [Source:HGNC Symbol;Acc:HGNC:2665] |
| ENSG00000135046 | ANXA1 | 2.373773519 | 1.28E-12 | annexin A1 [Source:HGNC Symbol;Acc:HGNC:533] |
| ENSG00000100644 | HIF1A | 1.394561456 | 1.46E-12 | hypoxia inducible factor 1 subunit alpha [Source:HGNC Symbol;Acc:HGNC:4910] |
| ENSG00000254166 | CASC19 | 2.435243335 | 2.14E-12 | cancer susceptibility 19 [Source:HGNC Symbol;Acc:HGNC:49476] |
| ENSG00000281990 | IGHV1-69-2 | 23.75054551 | 2.32E-12 | immunoglobulin heavy variable 1-69-2 [Source:HGNC Symbol;Acc:HGNC:5562] |
| ENSG00000112559 | MDFI | 2.703410315 | 2.47E-12 | MyoD family inhibitor [Source:HGNC Symbol;Acc:HGNC:6967] |
| ENSG00000166401 | SERPINB8 | 1.60659801 | 5.57E-12 | serpin family B member 8 [Source:HGNC Symbol;Acc:HGNC:8952] |
| ENSG00000181104 | F2R | 1.584300124 | 5.77E-12 | coagulation factor II thrombin receptor [Source:HGNC Symbol;Acc:HGNC:3537] |
| ENSG00000130508 | PXDN | 1.643398303 | 7.28E-12 | peroxidasin [Source:HGNC Symbol;Acc:HGNC:14966] |
| ENSG00000137812 | KNL1 | 1.408764175 | 1.26E-11 | kinetochore scaffold 1 [Source:HGNC Symbol;Acc:HGNC:24054] |
| ENSG00000261371 | PECAM1 | 1.36148098 | 1.27E-11 | platelet and endothelial cell adhesion molecule 1 [Source:HGNC Symbol;Acc:HGNC:8823] |
| ENSG00000114346 | ECT2 | 1.356019018 | 1.29E-11 | epithelial cell transforming 2 [Source:HGNC Symbol;Acc:HGNC:3155] |
| ENSG00000135636 | DYSF | 1.834366222 | 1.55E-11 | dysferlin [Source:HGNC Symbol;Acc:HGNC:3097] |
| ENSG00000170525 | PFKFB3 | 2.162875343 | 2.13E-11 | 6-phosphofructo-2-kinase/fructose-2,6-biphosphatase 3 [Source:HGNC Symbol;Acc:HGNC:8874] |
| ENSG00000115919 | KYNU | 2.722304985 | 2.42E-11 | kynureninase [Source:HGNC Symbol;Acc:HGNC:6469] |
| ENSG00000248323 | LUCAT1 | 4.821705862 | 2.68E-11 | lung cancer associated transcript 1 [Source:HGNC Symbol;Acc:HGNC:48498] |
| ENSG00000134871 | COL4A2 | 1.66788573 | 3.40E-11 | collagen type IV alpha 2 chain [Source:HGNC Symbol;Acc:HGNC:2203] |
| ENSG00000080573 | COL5A3 | 1.356673179 | 4.81E-11 | collagen type V alpha 3 chain [Source:HGNC Symbol;Acc:HGNC:14864] |
| ENSG00000198959 | TGM2 | 2.163367722 | 4.94E-11 | transglutaminase 2 [Source:HGNC Symbol;Acc:HGNC:11778] |
| ENSG00000172965 | MIR4435-2HG | 1.83874913 | 7.37E-11 | MIR4435-2 host gene [Source:HGNC Symbol;Acc:HGNC:35163] |
| ENSG00000262406 | MMP12 | 3.360861915 | 1.03E-10 | matrix metallopeptidase 12 [Source:HGNC Symbol;Acc:HGNC:7158] |
| ENSG00000068366 | ACSL4 | 1.508240721 | 1.17E-10 | acyl-CoA synthetase long chain family member 4 [Source:HGNC Symbol;Acc:HGNC:3571] |
| ENSG00000139618 | BRCA2 | 1.569305359 | 1.20E-10 | BRCA2, DNA repair associated [Source:HGNC Symbol;Acc:HGNC:1101] |
| ENSG00000163359 | COL6A3 | 2.413762972 | 1.20E-10 | collagen type VI alpha 3 chain [Source:HGNC Symbol;Acc:HGNC:2213] |
| ENSG00000189377 | CXCL17 | 7.385660988 | 1.31E-10 | C-X-C motif chemokine ligand 17 [Source:HGNC Symbol;Acc:HGNC:19232] |
| ENSG00000127507 | ADGRE2 | 2.454871331 | 1.37E-10 | adhesion G protein-coupled receptor E2 [Source:HGNC Symbol;Acc:HGNC:3337] |
| ENSG00000161638 | ITGA5 | 1.61647621 | 1.76E-10 | integrin subunit alpha 5 [Source:HGNC Symbol;Acc:HGNC:6141] |
| ENSG00000128052 | KDR | 1.69304038 | 1.93E-10 | kinase insert domain receptor [Source:HGNC Symbol;Acc:HGNC:6307] |
| ENSG00000134824 | FADS2 | 2.746805961 | 2.14E-10 | fatty acid desaturase 2 [Source:HGNC Symbol;Acc:HGNC:3575] |
| ENSG00000167767 | KRT80 | 4.300478594 | 2.31E-10 | keratin 80 [Source:HGNC Symbol;Acc:HGNC:27056] |
| ENSG00000129048 | ACKR4 | 2.760707432 | 4.56E-10 | atypical chemokine receptor 4 [Source:HGNC Symbol;Acc:HGNC:1611] |
| ENSG00000134757 | DSG3 | 4.383773842 | 6.48E-10 | desmoglein 3 [Source:HGNC Symbol;Acc:HGNC:3050] |
| ENSG00000008513 | ST3GAL1 | 2.075102104 | 6.66E-10 | ST3 beta-galactoside alpha-2,3-sialyltransferase 1 [Source:HGNC Symbol;Acc:HGNC:10862] |
| ENSG00000163421 | PROK2 | 5.168304848 | 6.90E-10 | prokineticin 2 [Source:HGNC Symbol;Acc:HGNC:18455] |
| ENSG00000115461 | IGFBP5 | 2.430841924 | 7.48E-10 | insulin like growth factor binding protein 5 [Source:HGNC Symbol;Acc:HGNC:5474] |
| ENSG00000123496 | IL13RA2 | 4.645976164 | 9.52E-10 | interleukin 13 receptor subunit alpha 2 [Source:HGNC Symbol;Acc:HGNC:5975] |
| ENSG00000159167 | STC1 | 3.100584705 | 1.31E-09 | stanniocalcin 1 [Source:HGNC Symbol;Acc:HGNC:11373] |
| ENSG00000168209 | DDIT4 | 1.56376582 | 1.33E-09 | DNA damage inducible transcript 4 [Source:HGNC Symbol;Acc:HGNC:24944] |
| ENSG00000182871 | COL18A1 | 1.334227394 | 1.86E-09 | collagen type XVIII alpha 1 chain [Source:HGNC Symbol;Acc:HGNC:2195] |
| ENSG00000011426 | ANLN | 1.568822431 | 2.48E-09 | anillin actin binding protein [Source:HGNC Symbol;Acc:HGNC:14082] |
| ENSG00000111331 | OAS3 | 1.146966046 | 2.54E-09 | 2'-5'-oligoadenylate synthetase 3 [Source:HGNC Symbol;Acc:HGNC:8088] |
| ENSG00000167460 | TPM4 | 1.116566744 | 3.07E-09 | tropomyosin 4 [Source:HGNC Symbol;Acc:HGNC:12013] |
| ENSG00000151948 | GLT1D1 | 3.424502995 | 3.24E-09 | glycosyltransferase 1 domain containing 1 [Source:HGNC Symbol;Acc:HGNC:26483] |
| ENSG00000173852 | DPY19L1 | 1.308694547 | 4.61E-09 | dpy-19 like C-mannosyltransferase 1 [Source:HGNC Symbol;Acc:HGNC:22205] |
| ENSG00000062038 | CDH3 | 3.45154504 | 4.61E-09 | cadherin 3 [Source:HGNC Symbol;Acc:HGNC:1762] |
| ENSG00000135480 | KRT7 | 3.405155308 | 4.85E-09 | keratin 7 [Source:HGNC Symbol;Acc:HGNC:6445] |
| ENSG00000164932 | CTHRC1 | 1.911599127 | 6.87E-09 | collagen triple helix repeat containing 1 [Source:HGNC Symbol;Acc:HGNC:18831] |
| ENSG00000163507 | CIP2A | 1.378261475 | 7.12E-09 | cell proliferation regulating inhibitor of protein phosphatase 2A [Source:HGNC Symbol;Acc:HGNC:29302] |
| ENSG00000106366 | SERPINE1 | 2.695172883 | 8.59E-09 | serpin family E member 1 [Source:HGNC Symbol;Acc:HGNC:8583] |
| ENSG00000091137 | SLC26A4 | 2.977548114 | 9.92E-09 | solute carrier family 26 member 4 [Source:HGNC Symbol;Acc:HGNC:8818] |
| ENSG00000136689 | IL1RN | 2.68584511 | 1.09E-08 | interleukin 1 receptor antagonist [Source:HGNC Symbol;Acc:HGNC:6000] |
| ENSG00000166396 | SERPINB7 | 9.806984766 | 1.12E-08 | serpin family B member 7 [Source:HGNC Symbol;Acc:HGNC:13902] |
| ENSG00000255398 | HCAR3 | 4.9814301 | 1.13E-08 | hydroxycarboxylic acid receptor 3 [Source:HGNC Symbol;Acc:HGNC:16824] |
| ENSG00000167772 | ANGPTL4 | 2.591844451 | 1.40E-08 | angiopoietin like 4 [Source:HGNC Symbol;Acc:HGNC:16039] |
| ENSG00000077232 | DNAJC10 | 1.070396305 | 1.56E-08 | DnaJ heat shock protein family (Hsp40) member C10 [Source:HGNC Symbol;Acc:HGNC:24637] |
| ENSG00000270550 | IGHV3-30 | 3.357392838 | 1.69E-08 | immunoglobulin heavy variable 3-30 [Source:HGNC Symbol;Acc:HGNC:5591] |
| ENSG00000122641 | INHBA | 4.028149973 | 1.95E-08 | inhibin subunit beta A [Source:HGNC Symbol;Acc:HGNC:6066] |
| ENSG00000148773 | MKI67 | 1.444488067 | 2.56E-08 | marker of proliferation Ki-67 [Source:HGNC Symbol;Acc:HGNC:7107] |
| ENSG00000177409 | SAMD9L | 1.780710467 | 2.75E-08 | sterile alpha motif domain containing 9 like [Source:HGNC Symbol;Acc:HGNC:1349] |
| ENSG00000069482 | GAL | 3.962506742 | 3.09E-08 | galanin and GMAP prepropeptide [Source:HGNC Symbol;Acc:HGNC:4114] |
| ENSG00000162745 | OLFML2B | 1.795248696 | 3.23E-08 | olfactomedin like 2B [Source:HGNC Symbol;Acc:HGNC:24558] |
| ENSG00000012660 | ELOVL5 | 1.442842602 | 4.08E-08 | ELOVL fatty acid elongase 5 [Source:HGNC Symbol;Acc:HGNC:21308] |
| ENSG00000065833 | ME1 | 1.84069287 | 4.08E-08 | malic enzyme 1 [Source:HGNC Symbol;Acc:HGNC:6983] |
| ENSG00000166130 | IKBIP | 1.221630326 | 4.75E-08 | IKBKB interacting protein [Source:HGNC Symbol;Acc:HGNC:26430] |
| ENSG00000166840 | GLYATL1 | 2.750913052 | 4.75E-08 | glycine-N-acyltransferase like 1 [Source:HGNC Symbol;Acc:HGNC:30519] |
| ENSG00000128918 | ALDH1A2 | 2.689903469 | 4.75E-08 | aldehyde dehydrogenase 1 family member A2 [Source:HGNC Symbol;Acc:HGNC:15472] |
| ENSG00000058085 | LAMC2 | 2.161369236 | 4.75E-08 | laminin subunit gamma 2 [Source:HGNC Symbol;Acc:HGNC:6493] |
| ENSG00000119535 | CSF3R | 2.511396709 | 4.77E-08 | colony stimulating factor 3 receptor [Source:HGNC Symbol;Acc:HGNC:2439] |
| ENSG00000156802 | ATAD2 | 1.213311083 | 4.98E-08 | ATPase family, AAA domain containing 2 [Source:HGNC Symbol;Acc:HGNC:30123] |
| ENSG00000138778 | CENPE | 1.291201516 | 4.99E-08 | centromere protein E [Source:HGNC Symbol;Acc:HGNC:1856] |
| ENSG00000118473 | SGIP1 | 2.107535377 | 5.31E-08 | SH3 domain GRB2 like endophilin interacting protein 1 [Source:HGNC Symbol;Acc:HGNC:25412] |
| ENSG00000179776 | CDH5 | 1.316183248 | 5.53E-08 | cadherin 5 [Source:HGNC Symbol;Acc:HGNC:1764] |
| ENSG00000104415 | WISP1 | 3.165451213 | 6.02E-08 | WNT1 inducible signaling pathway protein 1 [Source:HGNC Symbol;Acc:HGNC:12769] |
| ENSG00000188263 | IL17REL | 3.210971819 | 6.02E-08 | interleukin 17 receptor E like [Source:HGNC Symbol;Acc:HGNC:33808] |
| ENSG00000078098 | FAP | 3.025237881 | 6.10E-08 | fibroblast activation protein alpha [Source:HGNC Symbol;Acc:HGNC:3590] |
| ENSG00000186871 | ERCC6L | 1.449971318 | 6.58E-08 | ERCC excision repair 6 like, spindle assembly checkpoint helicase [Source:HGNC Symbol;Acc:HGNC:20794] |
| ENSG00000014257 | ACPP | 1.900720535 | 6.91E-08 | acid phosphatase, prostate [Source:HGNC Symbol;Acc:HGNC:125] |
| ENSG00000140678 | ITGAX | 1.67854849 | 7.44E-08 | integrin subunit alpha X [Source:HGNC Symbol;Acc:HGNC:6152] |
| ENSG00000128595 | CALU | 1.147869131 | 8.05E-08 | calumenin [Source:HGNC Symbol;Acc:HGNC:1458] |
| ENSG00000137699 | TRIM29 | 2.571562483 | 9.20E-08 | tripartite motif containing 29 [Source:HGNC Symbol;Acc:HGNC:17274] |
| ENSG00000165490 | DDIAS | 1.483538805 | 9.24E-08 | DNA damage induced apoptosis suppressor [Source:HGNC Symbol;Acc:HGNC:26351] |
| ENSG00000157551 | KCNJ15 | 3.474194003 | 9.68E-08 | potassium voltage-gated channel subfamily J member 15 [Source:HGNC Symbol;Acc:HGNC:6261] |
| ENSG00000229644 | NAMPTP1 | 1.501053537 | 1.06E-07 | nicotinamide phosphoribosyltransferase pseudogene 1 [Source:HGNC Symbol;Acc:HGNC:17633] |
| ENSG00000064989 | CALCRL | 1.732638253 | 1.23E-07 | calcitonin receptor like receptor [Source:HGNC Symbol;Acc:HGNC:16709] |
| ENSG00000130052 | STARD8 | 1.411692763 | 1.25E-07 | StAR related lipid transfer domain containing 8 [Source:HGNC Symbol;Acc:HGNC:19161] |
| ENSG00000164220 | F2RL2 | 2.709042579 | 1.39E-07 | coagulation factor II thrombin receptor like 2 [Source:HGNC Symbol;Acc:HGNC:3539] |
| ENSG00000206073 | SERPINB4 | 9.096793988 | 1.39E-07 | serpin family B member 4 [Source:HGNC Symbol;Acc:HGNC:10570] |
| ENSG00000151468 | CCDC3 | 2.028539524 | 1.47E-07 | coiled-coil domain containing 3 [Source:HGNC Symbol;Acc:HGNC:23813] |
| ENSG00000121361 | KCNJ8 | 2.016018923 | 1.74E-07 | potassium voltage-gated channel subfamily J member 8 [Source:HGNC Symbol;Acc:HGNC:6269] |
| ENSG00000173821 | RNF213 | 1.270508774 | 2.20E-07 | ring finger protein 213 [Source:HGNC Symbol;Acc:HGNC:14539] |
| ENSG00000172927 | MYEOV | 2.355734556 | 2.35E-07 | myeloma overexpressed [Source:HGNC Symbol;Acc:HGNC:7563] |
| ENSG00000151726 | ACSL1 | 1.059230147 | 2.36E-07 | acyl-CoA synthetase long chain family member 1 [Source:HGNC Symbol;Acc:HGNC:3569] |
| ENSG00000172016 | REG3A | 7.078002853 | 2.38E-07 | regenerating family member 3 alpha [Source:HGNC Symbol;Acc:HGNC:8601] |
| ENSG00000173706 | HEG1 | 1.621537623 | 2.50E-07 | heart development protein with EGF like domains 1 [Source:HGNC Symbol;Acc:HGNC:29227] |
| ENSG00000215182 | MUC5AC | 7.005152777 | 2.56E-07 | mucin 5AC, oligomeric mucus/gel-forming [Source:HGNC Symbol;Acc:HGNC:7515] |
| ENSG00000225614 | ZNF469 | 2.07172378 | 2.62E-07 | zinc finger protein 469 [Source:HGNC Symbol;Acc:HGNC:23216] |
| ENSG00000112414 | ADGRG6 | 2.253099974 | 2.63E-07 | adhesion G protein-coupled receptor G6 [Source:HGNC Symbol;Acc:HGNC:13841] |
| ENSG00000137673 | MMP7 | 5.764918213 | 2.63E-07 | matrix metallopeptidase 7 [Source:HGNC Symbol;Acc:HGNC:7174] |
| ENSG00000134851 | TMEM165 | 1.02032853 | 2.72E-07 | transmembrane protein 165 [Source:HGNC Symbol;Acc:HGNC:30760] |
| ENSG00000142748 | FCN3 | 3.399615732 | 2.80E-07 | ficolin 3 [Source:HGNC Symbol;Acc:HGNC:3625] |
| ENSG00000151012 | SLC7A11 | 2.367431402 | 2.83E-07 | solute carrier family 7 member 11 [Source:HGNC Symbol;Acc:HGNC:11059] |
| ENSG00000168502 | MTCL1 | 1.926375996 | 2.84E-07 | microtubule crosslinking factor 1 [Source:HGNC Symbol;Acc:HGNC:29121] |
| ENSG00000144452 | ABCA12 | 5.113111748 | 2.85E-07 | ATP binding cassette subfamily A member 12 [Source:HGNC Symbol;Acc:HGNC:14637] |
| ENSG00000169469 | SPRR1B | 8.348941799 | 2.89E-07 | small proline rich protein 1B [Source:HGNC Symbol;Acc:HGNC:11260] |
| ENSG00000117724 | CENPF | 1.189541996 | 2.91E-07 | centromere protein F [Source:HGNC Symbol;Acc:HGNC:1857] |
| ENSG00000076706 | MCAM | 1.349901256 | 3.42E-07 | melanoma cell adhesion molecule [Source:HGNC Symbol;Acc:HGNC:6934] |
| ENSG00000173193 | PARP14 | 1.107477031 | 3.42E-07 | poly(ADP-ribose) polymerase family member 14 [Source:HGNC Symbol;Acc:HGNC:29232] |
| ENSG00000042062 | RIPOR3 | 1.017699611 | 3.44E-07 | RIPOR family member 3 [Source:HGNC Symbol;Acc:HGNC:16168] |
| ENSG00000163638 | ADAMTS9 | 1.636274683 | 3.62E-07 | ADAM metallopeptidase with thrombospondin type 1 motif 9 [Source:HGNC Symbol;Acc:HGNC:13202] |
| ENSG00000134193 | REG4 | 4.097846765 | 3.89E-07 | regenerating family member 4 [Source:HGNC Symbol;Acc:HGNC:22977] |
| ENSG00000175445 | LPL | 2.530135018 | 4.09E-07 | lipoprotein lipase [Source:HGNC Symbol;Acc:HGNC:6677] |
| ENSG00000164294 | GPX8 | 1.453036387 | 4.28E-07 | glutathione peroxidase 8 (putative) [Source:HGNC Symbol;Acc:HGNC:33100] |
| ENSG00000122863 | CHST3 | 2.179772232 | 4.62E-07 | carbohydrate sulfotransferase 3 [Source:HGNC Symbol;Acc:HGNC:1971] |
| ENSG00000113140 | SPARC | 1.060098665 | 4.80E-07 | secreted protein acidic and cysteine rich [Source:HGNC Symbol;Acc:HGNC:11219] |
| ENSG00000123473 | STIL | 1.031694146 | 4.95E-07 | STIL, centriolar assembly protein [Source:HGNC Symbol;Acc:HGNC:10879] |
| ENSG00000168615 | ADAM9 | 1.360142685 | 5.26E-07 | ADAM metallopeptidase domain 9 [Source:HGNC Symbol;Acc:HGNC:216] |
| ENSG00000116962 | NID1 | 1.564110696 | 5.79E-07 | nidogen 1 [Source:HGNC Symbol;Acc:HGNC:7821] |
| ENSG00000090924 | PLEKHG2 | 1.375514701 | 6.41E-07 | pleckstrin homology and RhoGEF domain containing G2 [Source:HGNC Symbol;Acc:HGNC:29515] |
| ENSG00000242472 | IGHJ5 | 2.333994863 | 6.50E-07 | immunoglobulin heavy joining 5 [Source:HGNC Symbol;Acc:HGNC:5539] |
| ENSG00000131042 | LILRB2 | 1.524959461 | 6.91E-07 | leukocyte immunoglobulin like receptor B2 [Source:HGNC Symbol;Acc:HGNC:6606] |
| ENSG00000134317 | GRHL1 | 2.409701575 | 8.20E-07 | grainyhead like transcription factor 1 [Source:HGNC Symbol;Acc:HGNC:17923] |
| ENSG00000101187 | SLCO4A1 | 1.055091538 | 8.59E-07 | solute carrier organic anion transporter family member 4A1 [Source:HGNC Symbol;Acc:HGNC:10953] |
| ENSG00000106178 | CCL24 | 2.201771701 | 8.67E-07 | C-C motif chemokine ligand 24 [Source:HGNC Symbol;Acc:HGNC:10623] |
| ENSG00000148175 | STOM | 1.489377503 | 9.25E-07 | stomatin [Source:HGNC Symbol;Acc:HGNC:3383] |
| ENSG00000131389 | SLC6A6 | 1.641811732 | 9.73E-07 | solute carrier family 6 member 6 [Source:HGNC Symbol;Acc:HGNC:11052] |
| ENSG00000057149 | SERPINB3 | 9.617874135 | 1.01E-06 | serpin family B member 3 [Source:HGNC Symbol;Acc:HGNC:10569] |
| ENSG00000114554 | PLXNA1 | 1.077535207 | 1.03E-06 | plexin A1 [Source:HGNC Symbol;Acc:HGNC:9099] |
| ENSG00000102265 | TIMP1 | 1.552051626 | 1.05E-06 | TIMP metallopeptidase inhibitor 1 [Source:HGNC Symbol;Acc:HGNC:11820] |
| ENSG00000096968 | JAK2 | 1.215226618 | 1.12E-06 | Janus kinase 2 [Source:HGNC Symbol;Acc:HGNC:6192] |
| ENSG00000066279 | ASPM | 1.459411823 | 1.13E-06 | abnormal spindle microtubule assembly [Source:HGNC Symbol;Acc:HGNC:19048] |
| ENSG00000108821 | COL1A1 | 1.543483333 | 1.16E-06 | collagen type I alpha 1 chain [Source:HGNC Symbol;Acc:HGNC:2197] |
| ENSG00000005102 | MEOX1 | 1.371887962 | 1.20E-06 | mesenchyme homeobox 1 [Source:HGNC Symbol;Acc:HGNC:7013] |
| ENSG00000186638 | KIF24 | 1.161313115 | 1.21E-06 | kinesin family member 24 [Source:HGNC Symbol;Acc:HGNC:19916] |
| ENSG00000123838 | C4BPA | 4.095085726 | 1.24E-06 | complement component 4 binding protein alpha [Source:HGNC Symbol;Acc:HGNC:1325] |
| ENSG00000120708 | TGFBI | 1.260411669 | 1.39E-06 | transforming growth factor beta induced [Source:HGNC Symbol;Acc:HGNC:11771] |
| ENSG00000180871 | CXCR2 | 3.469508511 | 1.39E-06 | C-X-C motif chemokine receptor 2 [Source:HGNC Symbol;Acc:HGNC:6027] |
| ENSG00000128512 | DOCK4 | 1.19079866 | 1.46E-06 | dedicator of cytokinesis 4 [Source:HGNC Symbol;Acc:HGNC:19192] |
| ENSG00000070882 | OSBPL3 | 1.268685064 | 1.63E-06 | oxysterol binding protein like 3 [Source:HGNC Symbol;Acc:HGNC:16370] |
| ENSG00000149428 | HYOU1 | 1.000239344 | 1.64E-06 | hypoxia up-regulated 1 [Source:HGNC Symbol;Acc:HGNC:16931] |
| ENSG00000211950 | IGHV1-24 | 3.822704889 | 1.76E-06 | immunoglobulin heavy variable 1-24 [Source:HGNC Symbol;Acc:HGNC:5551] |
| ENSG00000221947 | XKR9 | 3.180931703 | 1.85E-06 | XK related 9 [Source:HGNC Symbol;Acc:HGNC:20937] |
| ENSG00000054938 | CHRDL2 | 3.164787086 | 2.00E-06 | chordin like 2 [Source:HGNC Symbol;Acc:HGNC:24168] |
| ENSG00000178726 | THBD | 1.456609837 | 2.05E-06 | thrombomodulin [Source:HGNC Symbol;Acc:HGNC:11784] |
| ENSG00000102359 | SRPX2 | 1.446872341 | 2.14E-06 | sushi repeat containing protein X-linked 2 [Source:HGNC Symbol;Acc:HGNC:30668] |
| ENSG00000145623 | OSMR | 1.672495345 | 2.17E-06 | oncostatin M receptor [Source:HGNC Symbol;Acc:HGNC:8507] |
| ENSG00000065328 | MCM10 | 1.470578086 | 2.31E-06 | minichromosome maintenance 10 replication initiation factor [Source:HGNC Symbol;Acc:HGNC:18043] |
| novel.115 | - | 2.258620519 | 2.33E-06 | PF13900:Putative domain of unknown function |
| ENSG00000064692 | SNCAIP | 1.697759293 | 2.33E-06 | synuclein alpha interacting protein [Source:HGNC Symbol;Acc:HGNC:11139] |
| ENSG00000240563 | L1TD1 | 2.164088482 | 2.36E-06 | LINE1 type transposase domain containing 1 [Source:HGNC Symbol;Acc:HGNC:25595] |
| ENSG00000258227 | CLEC5A | 3.01475376 | 2.40E-06 | C-type lectin domain containing 5A [Source:HGNC Symbol;Acc:HGNC:2054] |
| ENSG00000122861 | PLAU | 1.635945384 | 2.57E-06 | plasminogen activator, urokinase [Source:HGNC Symbol;Acc:HGNC:9052] |
| ENSG00000254087 | LYN | 1.191706359 | 2.66E-06 | LYN proto-oncogene, Src family tyrosine kinase [Source:HGNC Symbol;Acc:HGNC:6735] |
| ENSG00000165376 | CLDN2 | 4.264147989 | 2.75E-06 | claudin 2 [Source:HGNC Symbol;Acc:HGNC:2041] |
| ENSG00000138160 | KIF11 | 1.196160013 | 2.77E-06 | kinesin family member 11 [Source:HGNC Symbol;Acc:HGNC:6388] |
| ENSG00000120708 | TGFBI | 1.260411669 | 1.39E-06 | transforming growth factor beta induced [Source:HGNC Symbol;Acc:HGNC:11771] |
| ENSG00000198018 | ENTPD7 | 1.487679345 | 2.79E-06 | ectonucleoside triphosphate diphosphohydrolase 7 [Source:HGNC Symbol;Acc:HGNC:19745] |
| ENSG00000156219 | ART3 | 2.13354858 | 2.83E-06 | ADP-ribosyltransferase 3 [Source:HGNC Symbol;Acc:HGNC:725] |
| ENSG00000103888 | CEMIP | 1.902837846 | 3.15E-06 | cell migration inducing hyaluronidase 1 [Source:HGNC Symbol;Acc:HGNC:29213] |
| ENSG00000109805 | NCAPG | 1.326260302 | 3.15E-06 | non-SMC condensin I complex subunit G [Source:HGNC Symbol;Acc:HGNC:24304] |
| ENSG00000198814 | GK | 1.243532176 | 3.21E-06 | glycerol kinase [Source:HGNC Symbol;Acc:HGNC:4289] |
| ENSG00000184661 | CDCA2 | 1.415471866 | 3.29E-06 | cell division cycle associated 2 [Source:HGNC Symbol;Acc:HGNC:14623] |
| ENSG00000018280 | SLC11A1 | 1.95149061 | 3.30E-06 | solute carrier family 11 member 1 [Source:HGNC Symbol;Acc:HGNC:10907] |
| ENSG00000204262 | COL5A2 | 1.474955277 | 3.66E-06 | collagen type V alpha 2 chain [Source:HGNC Symbol;Acc:HGNC:2210] |
| ENSG00000128039 | SRD5A3 | 1.678173573 | 3.71E-06 | steroid 5 alpha-reductase 3 [Source:HGNC Symbol;Acc:HGNC:25812] |
| ENSG00000122188 | LAX1 | 2.080840421 | 3.71E-06 | lymphocyte transmembrane adaptor 1 [Source:HGNC Symbol;Acc:HGNC:26005] |
| ENSG00000198829 | SUCNR1 | 3.501055057 | 3.85E-06 | succinate receptor 1 [Source:HGNC Symbol;Acc:HGNC:4542] |
| ENSG00000138180 | CEP55 | 1.460478894 | 3.97E-06 | centrosomal protein 55 [Source:HGNC Symbol;Acc:HGNC:1161] |
| ENSG00000139734 | DIAPH3 | 1.39743242 | 4.03E-06 | diaphanous related formin 3 [Source:HGNC Symbol;Acc:HGNC:15480] |
| ENSG00000169607 | CKAP2L | 1.129633764 | 4.11E-06 | cytoskeleton associated protein 2 like [Source:HGNC Symbol;Acc:HGNC:26877] |
| ENSG00000100003 | SEC14L2 | 1.662089308 | 4.52E-06 | SEC14 like lipid binding 2 [Source:HGNC Symbol;Acc:HGNC:10699] |
| ENSG00000088882 | CPXM1 | 1.523031666 | 4.59E-06 | carboxypeptidase X, M14 family member 1 [Source:HGNC Symbol;Acc:HGNC:15771] |
| ENSG00000138346 | DNA2 | 1.051546886 | 4.59E-06 | DNA replication helicase/nuclease 2 [Source:HGNC Symbol;Acc:HGNC:2939] |
| ENSG00000176170 | SPHK1 | 1.50514614 | 4.59E-06 | sphingosine kinase 1 [Source:HGNC Symbol;Acc:HGNC:11240] |
| ENSG00000182782 | HCAR2 | 3.304086554 | 4.68E-06 | hydroxycarboxylic acid receptor 2 [Source:HGNC Symbol;Acc:HGNC:24827] |
| ENSG00000169908 | TM4SF1 | 2.025909233 | 5.00E-06 | transmembrane 4 L six family member 1 [Source:HGNC Symbol;Acc:HGNC:11853] |
| ENSG00000187583 | PLEKHN1 | 1.845102333 | 5.01E-06 | pleckstrin homology domain containing N1 [Source:HGNC Symbol;Acc:HGNC:25284] |
| ENSG00000140859 | KIFC3 | 1.210822046 | 5.25E-06 | kinesin family member C3 [Source:HGNC Symbol;Acc:HGNC:6326] |
| ENSG00000092853 | CLSPN | 1.681394255 | 5.31E-06 | claspin [Source:HGNC Symbol;Acc:HGNC:19715] |
| ENSG00000090376 | IRAK3 | 1.627940105 | 5.32E-06 | interleukin 1 receptor associated kinase 3 [Source:HGNC Symbol;Acc:HGNC:17020] |
| ENSG00000105835 | NAMPT | 1.246956853 | 5.33E-06 | nicotinamide phosphoribosyltransferase [Source:HGNC Symbol;Acc:HGNC:30092] |
| ENSG00000102755 | FLT1 | 1.313562985 | 5.65E-06 | fms related tyrosine kinase 1 [Source:HGNC Symbol;Acc:HGNC:3763] |
| ENSG00000145423 | SFRP2 | 5.561399839 | 5.66E-06 | secreted frizzled related protein 2 [Source:HGNC Symbol;Acc:HGNC:10777] |
| ENSG00000124875 | CXCL6 | 2.564351641 | 5.68E-06 | C-X-C motif chemokine ligand 6 [Source:HGNC Symbol;Acc:HGNC:10643] |
| ENSG00000133985 | TTC9 | 1.739271966 | 5.89E-06 | tetratricopeptide repeat domain 9 [Source:HGNC Symbol;Acc:HGNC:20267] |
| ENSG00000147689 | FAM83A | 3.793680982 | 5.92E-06 | family with sequence similarity 83 member A [Source:HGNC Symbol;Acc:HGNC:28210] |
| ENSG00000130164 | LDLR | 1.421743677 | 6.31E-06 | low density lipoprotein receptor [Source:HGNC Symbol;Acc:HGNC:6547] |
| ENSG00000131747 | TOP2A | 1.028460738 | 6.73E-06 | DNA topoisomerase II alpha [Source:HGNC Symbol;Acc:HGNC:11989] |
| ENSG00000134827 | TCN1 | 7.060280493 | 6.87E-06 | transcobalamin 1 [Source:HGNC Symbol;Acc:HGNC:11652] |
| ENSG00000163221 | S100A12 | 4.779068114 | 7.09E-06 | S100 calcium binding protein A12 [Source:HGNC Symbol;Acc:HGNC:10489] |
| ENSG00000173535 | TNFRSF10C | 2.626942402 | 7.11E-06 | TNF receptor superfamily member 10c [Source:HGNC Symbol;Acc:HGNC:11906] |
| ENSG00000135919 | SERPINE2 | 1.302363097 | 7.31E-06 | serpin family E member 2 [Source:HGNC Symbol;Acc:HGNC:8951] |
| ENSG00000136231 | IGF2BP3 | 4.264049401 | 7.68E-06 | insulin like growth factor 2 mRNA binding protein 3 [Source:HGNC Symbol;Acc:HGNC:28868] |
| ENSG00000164171 | ITGA2 | 2.21105903 | 7.72E-06 | integrin subunit alpha 2 [Source:HGNC Symbol;Acc:HGNC:6137] |
| ENSG00000160883 | HK3 | 1.449012201 | 8.36E-06 | hexokinase 3 [Source:HGNC Symbol;Acc:HGNC:4925] |
| ENSG00000004700 | RECQL | 1.483613937 | 9.01E-06 | RecQ like helicase [Source:HGNC Symbol;Acc:HGNC:9948] |
| ENSG00000144810 | COL8A1 | 1.928625138 | 9.07E-06 | collagen type VIII alpha 1 chain [Source:HGNC Symbol;Acc:HGNC:2215] |
| ENSG00000275063 | AC233755.1 | 10.1566992 | 9.35E-06 | immunoglobulin heavy variable 4-38-2-like [Source:NCBI gene;Acc:102723407] |
| ENSG00000163430 | FSTL1 | 1.030651752 | 9.35E-06 | follistatin like 1 [Source:HGNC Symbol;Acc:HGNC:3972] |
| ENSG00000113615 | SEC24A | 1.114561271 | 9.82E-06 | SEC24 homolog A, COPII coat complex component [Source:HGNC Symbol;Acc:HGNC:10703] |
| ENSG00000182885 | ADGRG3 | 2.320784227 | 1.00E-05 | adhesion G protein-coupled receptor G3 [Source:HGNC Symbol;Acc:HGNC:13728] |
| ENSG00000211651 | IGLV1-44 | 2.303625405 | 1.02E-05 | immunoglobulin lambda variable 1-44 [Source:HGNC Symbol;Acc:HGNC:5879] |
| ENSG00000054967 | RELT | 1.422676602 | 1.02E-05 | RELT, TNF receptor [Source:HGNC Symbol;Acc:HGNC:13764] |
| ENSG00000182158 | CREB3L2 | 1.045446715 | 1.04E-05 | cAMP responsive element binding protein 3 like 2 [Source:HGNC Symbol;Acc:HGNC:23720] |
| ENSG00000150961 | SEC24D | 1.10846787 | 1.05E-05 | SEC24 homolog D, COPII coat complex component [Source:HGNC Symbol;Acc:HGNC:10706] |
| ENSG00000171049 | FPR2 | 3.565257183 | 1.16E-05 | formyl peptide receptor 2 [Source:HGNC Symbol;Acc:HGNC:3827] |
| ENSG00000143641 | GALNT2 | 1.050547972 | 1.25E-05 | polypeptide N-acetylgalactosaminyltransferase 2 [Source:HGNC Symbol;Acc:HGNC:4124] |
| ENSG00000151014 | NOCT | 1.216819813 | 1.25E-05 | nocturnin [Source:HGNC Symbol;Acc:HGNC:14254] |
| ENSG00000183018 | SPNS2 | 2.060577307 | 1.27E-05 | sphingolipid transporter 2 [Source:HGNC Symbol;Acc:HGNC:26992] |
| ENSG00000211965 | IGHV3-49 | 1.881053304 | 1.27E-05 | immunoglobulin heavy variable 3-49 [Source:HGNC Symbol;Acc:HGNC:5607] |
| ENSG00000185479 | KRT6B | 4.039192215 | 1.30E-05 | keratin 6B [Source:HGNC Symbol;Acc:HGNC:6444] |
| ENSG00000175592 | FOSL1 | 2.731006833 | 1.31E-05 | FOS like 1, AP-1 transcription factor subunit [Source:HGNC Symbol;Acc:HGNC:13718] |
| ENSG00000204291 | COL15A1 | 1.298504702 | 1.34E-05 | collagen type XV alpha 1 chain [Source:HGNC Symbol;Acc:HGNC:2192] |
| ENSG00000211962 | IGHV1-46 | 2.331012423 | 1.44E-05 | immunoglobulin heavy variable 1-46 [Source:HGNC Symbol;Acc:HGNC:5554] |
| ENSG00000145386 | CCNA2 | 1.249434847 | 1.45E-05 | cyclin A2 [Source:HGNC Symbol;Acc:HGNC:1578] |
| ENSG00000123684 | LPGAT1 | 1.046755 | 1.50E-05 | lysophosphatidylglycerol acyltransferase 1 [Source:HGNC Symbol;Acc:HGNC:28985] |
| ENSG00000007908 | SELE | 2.571290736 | 1.61E-05 | selectin E [Source:HGNC Symbol;Acc:HGNC:10718] |
| ENSG00000114270 | COL7A1 | 1.599885959 | 1.61E-05 | collagen type VII alpha 1 chain [Source:HGNC Symbol;Acc:HGNC:2214] |
| ENSG00000019991 | HGF | 1.895389206 | 1.63E-05 | hepatocyte growth factor [Source:HGNC Symbol;Acc:HGNC:4893] |
| ENSG00000188487 | INSC | 1.4278449 | 1.63E-05 | INSC, spindle orientation adaptor protein [Source:HGNC Symbol;Acc:HGNC:33116] |
| ENSG00000104213 | PDGFRL | 1.543715399 | 1.63E-05 | platelet derived growth factor receptor like [Source:HGNC Symbol;Acc:HGNC:8805] |
| ENSG00000173156 | RHOD | 1.65105898 | 1.68E-05 | ras homolog family member D [Source:HGNC Symbol;Acc:HGNC:670] |
| ENSG00000237111 | IGHJ3P | 2.033410227 | 1.81E-05 | immunoglobulin heavy joining 3P (pseudogene) [Source:HGNC Symbol;Acc:HGNC:5537] |
| ENSG00000116191 | RALGPS2 | 1.068755915 | 1.85E-05 | Ral GEF with PH domain and SH3 binding motif 2 [Source:HGNC Symbol;Acc:HGNC:30279] |
| ENSG00000118946 | PCDH17 | 1.725900448 | 1.85E-05 | protocadherin 17 [Source:HGNC Symbol;Acc:HGNC:14267] |
| ENSG00000136378 | ADAMTS7 | 1.248708761 | 1.91E-05 | ADAM metallopeptidase with thrombospondin type 1 motif 7 [Source:HGNC Symbol;Acc:HGNC:223] |
| ENSG00000171517 | LPAR3 | 1.459091964 | 1.91E-05 | lysophosphatidic acid receptor 3 [Source:HGNC Symbol;Acc:HGNC:14298] |
| ENSG00000170801 | HTRA3 | 1.364406554 | 2.03E-05 | HtrA serine peptidase 3 [Source:HGNC Symbol;Acc:HGNC:30406] |
| ENSG00000183853 | KIRREL1 | 1.23916981 | 2.03E-05 | kirre like nephrin family adhesion molecule 1 [Source:HGNC Symbol;Acc:HGNC:15734] |
| novel.108 | - | 2.007502061 | 2.08E-05 | PF13900:Putative domain of unknown function |
| ENSG00000103257 | SLC7A5 | 1.792935034 | 2.12E-05 | solute carrier family 7 member 5 [Source:HGNC Symbol;Acc:HGNC:11063] |
| ENSG00000140937 | CDH11 | 1.054981448 | 2.14E-05 | cadherin 11 [Source:HGNC Symbol;Acc:HGNC:1750] |
| ENSG00000147065 | MSN | 1.252041718 | 2.21E-05 | moesin [Source:HGNC Symbol;Acc:HGNC:7373] |
| ENSG00000071242 | RPS6KA2 | 1.079813245 | 2.21E-05 | ribosomal protein S6 kinase A2 [Source:HGNC Symbol;Acc:HGNC:10431] |
| ENSG00000211966 | IGHV5-51 | 2.229580994 | 2.31E-05 | immunoglobulin heavy variable 5-51 [Source:HGNC Symbol;Acc:HGNC:5659] |
| ENSG00000071575 | TRIB2 | 1.643767041 | 2.38E-05 | tribbles pseudokinase 2 [Source:HGNC Symbol;Acc:HGNC:30809] |
| ENSG00000152669 | CCNO | 2.149028875 | 2.41E-05 | cyclin O [Source:HGNC Symbol;Acc:HGNC:18576] |
| ENSG00000159261 | CLDN14 | 3.702681089 | 2.54E-05 | claudin 14 [Source:HGNC Symbol;Acc:HGNC:2035] |
| ENSG00000143248 | RGS5 | 1.485139702 | 2.75E-05 | regulator of G protein signaling 5 [Source:HGNC Symbol;Acc:HGNC:10001] |
| ENSG00000135069 | PSAT1 | 1.891102405 | 2.78E-05 | phosphoserine aminotransferase 1 [Source:HGNC Symbol;Acc:HGNC:19129] |
| ENSG00000153317 | ASAP1 | 1.292191646 | 2.88E-05 | ArfGAP with SH3 domain, ankyrin repeat and PH domain 1 [Source:HGNC Symbol;Acc:HGNC:2720] |
| ENSG00000100078 | PLA2G3 | 3.614264433 | 2.94E-05 | phospholipase A2 group III [Source:HGNC Symbol;Acc:HGNC:17934] |
| ENSG00000177464 | GPR4 | 1.757011538 | 2.94E-05 | G protein-coupled receptor 4 [Source:HGNC Symbol;Acc:HGNC:4497] |
| ENSG00000112303 | VNN2 | 1.865735708 | 2.96E-05 | vanin 2 [Source:HGNC Symbol;Acc:HGNC:12706] |
| ENSG00000088002 | SULT2B1 | 1.450751378 | 3.00E-05 | sulfotransferase family 2B member 1 [Source:HGNC Symbol;Acc:HGNC:11459] |
| ENSG00000176971 | FIBIN | 2.819242117 | 3.01E-05 | fin bud initiation factor homolog [Source:HGNC Symbol;Acc:HGNC:33747] |
| ENSG00000162618 | ADGRL4 | 1.073417757 | 3.14E-05 | adhesion G protein-coupled receptor L4 [Source:HGNC Symbol;Acc:HGNC:20822] |
| ENSG00000138182 | KIF20B | 1.125855858 | 3.18E-05 | kinesin family member 20B [Source:HGNC Symbol;Acc:HGNC:7212] |
| ENSG00000109089 | CDR2L | 1.096485997 | 3.31E-05 | cerebellar degeneration related protein 2 like [Source:HGNC Symbol;Acc:HGNC:29999] |
| ENSG00000116285 | ERRFI1 | 1.467301554 | 3.31E-05 | ERBB receptor feedback inhibitor 1 [Source:HGNC Symbol;Acc:HGNC:18185] |
| ENSG00000106772 | PRUNE2 | 1.308196455 | 3.44E-05 | prune homolog 2 [Source:HGNC Symbol;Acc:HGNC:25209] |
| ENSG00000154217 | PITPNC1 | 1.075148894 | 3.45E-05 | phosphatidylinositol transfer protein cytoplasmic 1 [Source:HGNC Symbol;Acc:HGNC:21045] |
| ENSG00000164692 | COL1A2 | 1.191842521 | 3.47E-05 | collagen type I alpha 2 chain [Source:HGNC Symbol;Acc:HGNC:2198] |
| ENSG00000198826 | ARHGAP11A | 1.132401196 | 3.50E-05 | Rho GTPase activating protein 11A [Source:HGNC Symbol;Acc:HGNC:15783] |
| ENSG00000128641 | MYO1B | 1.126551497 | 3.52E-05 | myosin IB [Source:HGNC Symbol;Acc:HGNC:7596] |
| ENSG00000211896 | IGHG1 | 2.787141453 | 3.73E-05 | immunoglobulin heavy constant gamma 1 (G1m marker) [Source:HGNC Symbol;Acc:HGNC:5525] |
| ENSG00000134460 | IL2RA | 2.134277939 | 3.80E-05 | interleukin 2 receptor subunit alpha [Source:HGNC Symbol;Acc:HGNC:6008] |
| ENSG00000164211 | STARD4 | 1.107319538 | 3.81E-05 | StAR related lipid transfer domain containing 4 [Source:HGNC Symbol;Acc:HGNC:18058] |
| ENSG00000122966 | CIT | 1.05965137 | 3.81E-05 | citron rho-interacting serine/threonine kinase [Source:HGNC Symbol;Acc:HGNC:1985] |
| ENSG00000091879 | ANGPT2 | 1.661409741 | 3.82E-05 | angiopoietin 2 [Source:HGNC Symbol;Acc:HGNC:485] |
| ENSG00000168461 | RAB31 | 1.252539488 | 3.95E-05 | RAB31, member RAS oncogene family [Source:HGNC Symbol;Acc:HGNC:9771] |
| ENSG00000259863 | SH3RF3-AS1 | 2.3572602 | 4.04E-05 | SH3RF3 antisense RNA 1 [Source:HGNC Symbol;Acc:HGNC:44168] |
| ENSG00000087116 | ADAMTS2 | 1.341924634 | 4.05E-05 | ADAM metallopeptidase with thrombospondin type 1 motif 2 [Source:HGNC Symbol;Acc:HGNC:218] |
| ENSG00000166546 | BEAN1 | 3.164117323 | 4.29E-05 | brain expressed associated with NEDD4 1 [Source:HGNC Symbol;Acc:HGNC:24160] |
| ENSG00000172752 | COL6A5 | 2.793136544 | 4.34E-05 | collagen type VI alpha 5 chain [Source:HGNC Symbol;Acc:HGNC:26674] |
| ENSG00000049768 | FOXP3 | 1.807308533 | 4.36E-05 | forkhead box P3 [Source:HGNC Symbol;Acc:HGNC:6106] |
| ENSG00000120217 | CD274 | 1.75050894 | 4.38E-05 | CD274 molecule [Source:HGNC Symbol;Acc:HGNC:17635] |
| ENSG00000196611 | MMP1 | 3.594484713 | 4.40E-05 | matrix metallopeptidase 1 [Source:HGNC Symbol;Acc:HGNC:7155] |
| ENSG00000249992 | TMEM158 | 1.195148704 | 4.45E-05 | transmembrane protein 158 (gene/pseudogene) [Source:HGNC Symbol;Acc:HGNC:30293] |
| ENSG00000116329 | OPRD1 | 2.517263281 | 4.46E-05 | opioid receptor delta 1 [Source:HGNC Symbol;Acc:HGNC:8153] |
| ENSG00000183625 | CCR3 | 2.529087234 | 4.57E-05 | C-C motif chemokine receptor 3 [Source:HGNC Symbol;Acc:HGNC:1604] |
| ENSG00000177602 | HASPIN | 1.25087498 | 4.57E-05 | histone H3 associated protein kinase [Source:HGNC Symbol;Acc:HGNC:19682] |
| ENSG00000109790 | KLHL5 | 1.500617823 | 4.64E-05 | kelch like family member 5 [Source:HGNC Symbol;Acc:HGNC:6356] |
| ENSG00000163347 | CLDN1 | 2.412188457 | 4.66E-05 | claudin 1 [Source:HGNC Symbol;Acc:HGNC:2032] |
| ENSG00000006327 | TNFRSF12A | 1.592035675 | 4.66E-05 | TNF receptor superfamily member 12A [Source:HGNC Symbol;Acc:HGNC:18152] |
| ENSG00000176171 | BNIP3 | 1.011675549 | 4.78E-05 | BCL2 interacting protein 3 [Source:HGNC Symbol;Acc:HGNC:1084] |
| ENSG00000184985 | SORCS2 | 1.561117135 | 5.10E-05 | sortilin related VPS10 domain containing receptor 2 [Source:HGNC Symbol;Acc:HGNC:16698] |
| ENSG00000090382 | LYZ | 1.465129548 | 5.17E-05 | lysozyme [Source:HGNC Symbol;Acc:HGNC:6740] |
| ENSG00000150457 | LATS2 | 1.304734108 | 5.35E-05 | large tumor suppressor kinase 2 [Source:HGNC Symbol;Acc:HGNC:6515] |
| ENSG00000113721 | PDGFRB | 1.071594968 | 5.37E-05 | platelet derived growth factor receptor beta [Source:HGNC Symbol;Acc:HGNC:8804] |
| ENSG00000101255 | TRIB3 | 1.200418087 | 5.38E-05 | tribbles pseudokinase 3 [Source:HGNC Symbol;Acc:HGNC:16228] |
| ENSG00000143387 | CTSK | 1.336158518 | 5.46E-05 | cathepsin K [Source:HGNC Symbol;Acc:HGNC:2536] |
| ENSG00000103222 | ABCC1 | 1.106622527 | 5.57E-05 | ATP binding cassette subfamily C member 1 [Source:HGNC Symbol;Acc:HGNC:51] |
| ENSG00000132357 | CARD6 | 1.183483665 | 5.69E-05 | caspase recruitment domain family member 6 [Source:HGNC Symbol;Acc:HGNC:16394] |
| ENSG00000160862 | AZGP1 | 2.166958935 | 5.72E-05 | alpha-2-glycoprotein 1, zinc-binding [Source:HGNC Symbol;Acc:HGNC:910] |
| ENSG00000007933 | FMO3 | 3.254339069 | 5.79E-05 | flavin containing monooxygenase 3 [Source:HGNC Symbol;Acc:HGNC:3771] |
| ENSG00000181577 | C6orf223 | 3.58248522 | 5.80E-05 | chromosome 6 open reading frame 223 [Source:HGNC Symbol;Acc:HGNC:28692] |
| ENSG00000153395 | LPCAT1 | 1.490601897 | 5.82E-05 | lysophosphatidylcholine acyltransferase 1 [Source:HGNC Symbol;Acc:HGNC:25718] |
| ENSG00000136859 | ANGPTL2 | 1.314148289 | 6.26E-05 | angiopoietin like 2 [Source:HGNC Symbol;Acc:HGNC:490] |
| ENSG00000198535 | C2CD4A | 2.708124677 | 6.31E-05 | C2 calcium dependent domain containing 4A [Source:HGNC Symbol;Acc:HGNC:33627] |
| ENSG00000169474 | SPRR1A | 5.792716768 | 6.48E-05 | small proline rich protein 1A [Source:HGNC Symbol;Acc:HGNC:11259] |
| ENSG00000273962 | IGKV2-40 | 6.248944867 | 6.49E-05 | immunoglobulin kappa variable 2-40 [Source:HGNC Symbol;Acc:HGNC:5789] |
| ENSG00000151617 | EDNRA | 1.374864862 | 6.49E-05 | endothelin receptor type A [Source:HGNC Symbol;Acc:HGNC:3179] |
| ENSG00000242887 | IGHJ3 | 2.907060439 | 6.82E-05 | immunoglobulin heavy joining 3 [Source:HGNC Symbol;Acc:HGNC:5536] |
| ENSG00000023445 | BIRC3 | 1.508383698 | 6.87E-05 | baculoviral IAP repeat containing 3 [Source:HGNC Symbol;Acc:HGNC:591] |
| ENSG00000284946 | AC068831.7 | 1.320532766 | 6.88E-05 | novel transcript |
| ENSG00000102384 | CENPI | 1.383454888 | 6.90E-05 | centromere protein I [Source:HGNC Symbol;Acc:HGNC:3968] |
| ENSG00000197506 | SLC28A3 | 1.440202725 | 6.94E-05 | solute carrier family 28 member 3 [Source:HGNC Symbol;Acc:HGNC:16484] |
| ENSG00000113578 | FGF1 | 2.863598013 | 7.06E-05 | fibroblast growth factor 1 [Source:HGNC Symbol;Acc:HGNC:3665] |
| ENSG00000066294 | CD84 | 1.57308061 | 7.17E-05 | CD84 molecule [Source:HGNC Symbol;Acc:HGNC:1704] |
| ENSG00000164023 | SGMS2 | 1.176619115 | 7.20E-05 | sphingomyelin synthase 2 [Source:HGNC Symbol;Acc:HGNC:28395] |
| ENSG00000174705 | SH3PXD2B | 1.139249887 | 7.38E-05 | SH3 and PX domains 2B [Source:HGNC Symbol;Acc:HGNC:29242] |
| ENSG00000250899 | AC125807.2 | 1.400463753 | 7.88E-05 | novel transcript |
| ENSG00000135604 | STX11 | 1.477380647 | 7.88E-05 | syntaxin 11 [Source:HGNC Symbol;Acc:HGNC:11429] |
| ENSG00000121621 | KIF18A | 1.253627896 | 8.09E-05 | kinesin family member 18A [Source:HGNC Symbol;Acc:HGNC:29441] |
| ENSG00000168394 | TAP1 | 1.0196651 | 8.09E-05 | transporter 1, ATP binding cassette subfamily B member [Source:HGNC Symbol;Acc:HGNC:43] |
| ENSG00000166147 | FBN1 | 1.421364703 | 8.27E-05 | fibrillin 1 [Source:HGNC Symbol;Acc:HGNC:3603] |
| ENSG00000121594 | CD80 | 2.419764161 | 8.44E-05 | CD80 molecule [Source:HGNC Symbol;Acc:HGNC:1700] |
| ENSG00000211599 | IGKV5-2 | 2.759436561 | 8.92E-05 | immunoglobulin kappa variable 5-2 [Source:HGNC Symbol;Acc:HGNC:5835] |
| ENSG00000197405 | C5AR1 | 1.447870899 | 9.02E-05 | complement C5a receptor 1 [Source:HGNC Symbol;Acc:HGNC:1338] |
| ENSG00000211951 | IGHV2-26 | 2.536333065 | 9.04E-05 | immunoglobulin heavy variable 2-26 [Source:HGNC Symbol;Acc:HGNC:5575] |
| ENSG00000172243 | CLEC7A | 1.021078689 | 9.07E-05 | C-type lectin domain containing 7A [Source:HGNC Symbol;Acc:HGNC:14558] |
| ENSG00000136383 | ALPK3 | 1.699684622 | 9.24E-05 | alpha kinase 3 [Source:HGNC Symbol;Acc:HGNC:17574] |
| ENSG00000189233 | NUGGC | 1.296907987 | 9.32E-05 | nuclear GTPase, germinal center associated [Source:HGNC Symbol;Acc:HGNC:33550] |
| ENSG00000164542 | KIAA0895 | 1.391038297 | 9.37E-05 | KIAA0895 [Source:HGNC Symbol;Acc:HGNC:22206] |
| ENSG00000211959 | IGHV4-39 | 2.476026622 | 9.63E-05 | immunoglobulin heavy variable 4-39 [Source:HGNC Symbol;Acc:HGNC:5651] |
| ENSG00000116852 | KIF21B | 1.173424147 | 9.65E-05 | kinesin family member 21B [Source:HGNC Symbol;Acc:HGNC:29442] |
| ENSG00000189057 | FAM111B | 1.297280314 | 9.79E-05 | family with sequence similarity 111 member B [Source:HGNC Symbol;Acc:HGNC:24200] |
| ENSG00000114251 | WNT5A | 1.575866314 | 9.81E-05 | Wnt family member 5A [Source:HGNC Symbol;Acc:HGNC:12784] |
| ENSG00000111799 | COL12A1 | 2.373889743 | 9.94E-05 | collagen type XII alpha 1 chain [Source:HGNC Symbol;Acc:HGNC:2188] |
| ENSG00000134107 | BHLHE40 | 1.313427117 | 0.000104409 | basic helix-loop-helix family member e40 [Source:HGNC Symbol;Acc:HGNC:1046] |
| ENSG00000088340 | FER1L4 | 1.563815397 | 0.000106263 | fer-1 like family member 4, pseudogene [Source:HGNC Symbol;Acc:HGNC:15801] |
| ENSG00000074181 | NOTCH3 | 1.300124203 | 0.000107018 | notch 3 [Source:HGNC Symbol;Acc:HGNC:7883] |
| ENSG00000131355 | ADGRE3 | 2.888304883 | 0.000107497 | adhesion G protein-coupled receptor E3 [Source:HGNC Symbol;Acc:HGNC:23647] |
| ENSG00000244116 | IGKV2-28 | 1.850409252 | 0.000108839 | immunoglobulin kappa variable 2-28 [Source:HGNC Symbol;Acc:HGNC:5783] |
| ENSG00000164935 | DCSTAMP | 5.999950055 | 0.00010914 | dendrocyte expressed seven transmembrane protein [Source:HGNC Symbol;Acc:HGNC:18549] |
| ENSG00000065923 | SLC9A7 | 1.426195608 | 0.000110427 | solute carrier family 9 member A7 [Source:HGNC Symbol;Acc:HGNC:17123] |
| ENSG00000166845 | C18orf54 | 1.071071345 | 0.000112071 | chromosome 18 open reading frame 54 [Source:HGNC Symbol;Acc:HGNC:13796] |
| ENSG00000166073 | GPR176 | 1.370219749 | 0.000113269 | G protein-coupled receptor 176 [Source:HGNC Symbol;Acc:HGNC:32370] |
| ENSG00000128567 | PODXL | 1.108308302 | 0.000114807 | podocalyxin like [Source:HGNC Symbol;Acc:HGNC:9171] |
| novel.4 | - | 5.400868188 | 0.000117064 | PF13900:Putative domain of unknown function |
| ENSG00000135744 | AGT | 1.889866491 | 0.000121783 | angiotensinogen [Source:HGNC Symbol;Acc:HGNC:333] |
| ENSG00000100368 | CSF2RB | 1.39642546 | 0.000122894 | colony stimulating factor 2 receptor beta common subunit [Source:HGNC Symbol;Acc:HGNC:2436] |
| ENSG00000112984 | KIF20A | 1.262098173 | 0.000122894 | kinesin family member 20A [Source:HGNC Symbol;Acc:HGNC:9787] |
| ENSG00000188610 | FAM72B | 1.696552483 | 0.000125998 | family with sequence similarity 72 member B [Source:HGNC Symbol;Acc:HGNC:24805] |
| ENSG00000156970 | BUB1B | 1.134334857 | 0.000131075 | BUB1 mitotic checkpoint serine/threonine kinase B [Source:HGNC Symbol;Acc:HGNC:1149] |
| ENSG00000185480 | PARPBP | 1.134279746 | 0.000131334 | PARP1 binding protein [Source:HGNC Symbol;Acc:HGNC:26074] |
| ENSG00000130234 | ACE2 | 1.782597698 | 0.000136149 | angiotensin I converting enzyme 2 [Source:HGNC Symbol;Acc:HGNC:13557] |
| ENSG00000143226 | FCGR2A | 1.566807716 | 0.000136938 | Fc fragment of IgG receptor IIa [Source:HGNC Symbol;Acc:HGNC:3616] |
| ENSG00000163762 | TM4SF18 | 1.043178606 | 0.000141266 | transmembrane 4 L six family member 18 [Source:HGNC Symbol;Acc:HGNC:25181] |
| ENSG00000152413 | HOMER1 | 1.481384086 | 0.000142227 | homer scaffold protein 1 [Source:HGNC Symbol;Acc:HGNC:17512] |
| ENSG00000265107 | GJA5 | 2.079581982 | 0.000143839 | gap junction protein alpha 5 [Source:HGNC Symbol;Acc:HGNC:4279] |
| ENSG00000275896 | PRSS2 | 5.451978785 | 0.000144389 | serine protease 2 [Source:HGNC Symbol;Acc:HGNC:9483] |
| ENSG00000106415 | GLCCI1 | 1.091390174 | 0.000144389 | glucocorticoid induced 1 [Source:HGNC Symbol;Acc:HGNC:18713] |
| ENSG00000110427 | KIAA1549L | 3.538418998 | 0.000148579 | KIAA1549 like [Source:HGNC Symbol;Acc:HGNC:24836] |
| ENSG00000154451 | GBP5 | 1.714483839 | 0.000149179 | guanylate binding protein 5 [Source:HGNC Symbol;Acc:HGNC:19895] |
| ENSG00000182963 | GJC1 | 1.476654478 | 0.000150626 | gap junction protein gamma 1 [Source:HGNC Symbol;Acc:HGNC:4280] |
| ENSG00000198431 | TXNRD1 | 1.042849289 | 0.000152006 | thioredoxin reductase 1 [Source:HGNC Symbol;Acc:HGNC:12437] |
| ENSG00000169679 | BUB1 | 1.289293883 | 0.000152006 | BUB1 mitotic checkpoint serine/threonine kinase [Source:HGNC Symbol;Acc:HGNC:1148] |
| ENSG00000259230 | LINC02323 | 2.86935491 | 0.000153526 | long intergenic non-protein coding RNA 2323 [Source:HGNC Symbol;Acc:HGNC:53242] |
| ENSG00000197520 | FAM177B | 1.545208622 | 0.000156694 | family with sequence similarity 177 member B [Source:HGNC Symbol;Acc:HGNC:34395] |
| ENSG00000186407 | CD300E | 2.08702414 | 0.000158866 | CD300e molecule [Source:HGNC Symbol;Acc:HGNC:28874] |
| ENSG00000133048 | CHI3L1 | 4.122875828 | 0.000161097 | chitinase 3 like 1 [Source:HGNC Symbol;Acc:HGNC:1932] |
| ENSG00000162779 | AXDND1 | 2.191465322 | 0.000162711 | axonemal dynein light chain domain containing 1 [Source:HGNC Symbol;Acc:HGNC:26564] |
| ENSG00000277856 | AC233755.2 | 4.337049111 | 0.000164603 |  |
| ENSG00000240041 | IGHJ4 | 2.588165943 | 0.000164603 | immunoglobulin heavy joining 4 [Source:HGNC Symbol;Acc:HGNC:5538] |
| ENSG00000060558 | GNA15 | 1.490744661 | 0.000164624 | G protein subunit alpha 15 [Source:HGNC Symbol;Acc:HGNC:4383] |
| ENSG00000204301 | NOTCH4 | 1.015363587 | 0.000166282 | notch 4 [Source:HGNC Symbol;Acc:HGNC:7884] |
| ENSG00000183856 | IQGAP3 | 1.110001295 | 0.000167485 | IQ motif containing GTPase activating protein 3 [Source:HGNC Symbol;Acc:HGNC:20669] |
| ENSG00000175426 | PCSK1 | 1.656864163 | 0.000170573 | proprotein convertase subtilisin/kexin type 1 [Source:HGNC Symbol;Acc:HGNC:8743] |
| ENSG00000070190 | DAPP1 | 1.30604126 | 0.000171247 | dual adaptor of phosphotyrosine and 3-phosphoinositides 1 [Source:HGNC Symbol;Acc:HGNC:16500] |
| ENSG00000182853 | VMO1 | 1.324005047 | 0.000175275 | vitelline membrane outer layer 1 homolog [Source:HGNC Symbol;Acc:HGNC:30387] |
| ENSG00000138411 | HECW2 | 1.725763866 | 0.000181152 | HECT, C2 and WW domain containing E3 ubiquitin protein ligase 2 [Source:HGNC Symbol;Acc:HGNC:29853] |
| ENSG00000166123 | GPT2 | 1.146985553 | 0.000181567 | glutamic--pyruvic transaminase 2 [Source:HGNC Symbol;Acc:HGNC:18062] |
| ENSG00000115415 | STAT1 | 1.180270536 | 0.000181567 | signal transducer and activator of transcription 1 [Source:HGNC Symbol;Acc:HGNC:11362] |
| ENSG00000228168 | HNRNPA1P21 | 2.455320189 | 0.000190251 | heterogeneous nuclear ribonucleoprotein A1 pseudogene 21 [Source:HGNC Symbol;Acc:HGNC:39539] |
| ENSG00000197794 | IGKV7-3 | 2.845847108 | 0.000192397 | immunoglobulin kappa variable 7-3 (pseudogene) [Source:HGNC Symbol;Acc:HGNC:5839] |
| ENSG00000140534 | TICRR | 1.23826585 | 0.000193347 | TOPBP1 interacting checkpoint and replication regulator [Source:HGNC Symbol;Acc:HGNC:28704] |
| ENSG00000182492 | BGN | 1.298694504 | 0.000194424 | biglycan [Source:HGNC Symbol;Acc:HGNC:1044] |
| ENSG00000168386 | FILIP1L | 1.111132335 | 0.000196121 | filamin A interacting protein 1 like [Source:HGNC Symbol;Acc:HGNC:24589] |
| ENSG00000158186 | MRAS | 1.069584931 | 0.000203544 | muscle RAS oncogene homolog [Source:HGNC Symbol;Acc:HGNC:7227] |
| ENSG00000132182 | NUP210 | 1.078644058 | 0.000205522 | nucleoporin 210 [Source:HGNC Symbol;Acc:HGNC:30052] |
| ENSG00000137807 | KIF23 | 1.134038537 | 0.000209829 | kinesin family member 23 [Source:HGNC Symbol;Acc:HGNC:6392] |
| ENSG00000116701 | NCF2 | 1.495635594 | 0.000211343 | neutrophil cytosolic factor 2 [Source:HGNC Symbol;Acc:HGNC:7661] |
| ENSG00000164379 | FOXQ1 | 2.07362225 | 0.000213849 | forkhead box Q1 [Source:HGNC Symbol;Acc:HGNC:20951] |
| ENSG00000102962 | CCL22 | 2.141460804 | 0.000213849 | C-C motif chemokine ligand 22 [Source:HGNC Symbol;Acc:HGNC:10621] |
