# Supplementary material for: Transcriptomic analyses of treatment-naïve pediatric ulcerative colitis patients and exploration of underlying disease pathogenesis
Source: J Transl Med. 2023 Jan 16;21:30. doi: 10.1186/s12967-023-03881-6 (PMC9843999; doi:10.1186/s12967-023-03881-6)
Supplement: Supplementary file 4 — Additional file 4: Table S4. Top 30 gene ontology distributions of up-expressed genes between UC group and control group. [file 12967_2023_3881_MOESM4_ESM.doc]

**Table S4. Top 30 gene ontology distributions of up-expressed genes between UC group and control group**

| GO accession | Description | Count | Some of up Gene name | Over represented p-Value |
| --- | --- | --- | --- | --- |
| GO:0002455 | humoral immune response mediated by circulating immunoglobulin | 63 | CD55/IGHV1-69-2/IGHV3-30/C4BPA/IGHV1-24/AC233755.1/IGLV1-44/IGHV3-49/IGHV1-46/IGHV5-51/IGHG1/IGKV5-2/IGHV2-26/IGHV4-39/IGKV2-28/IGLV3-1/IGHV3-48/IGLV1-40/IGKV1-12/IGHV2-70/IGHV3-15/IGLV1-51/IGKV2D-28/IGKV2D-40/IGLV3-27/CFI/IGKV2-30/IGHV1-69/PTPRC/IGHV3-43/IGKV1-5/IGKV1-33/IGHV3-21/IGHV3-64/C4BPB/IGHV4-59/IGKV2D-30/IGHV3-74/IGLV2-14/IGHV1-18/IGHV3-7/IGKV3-15/HLA-DRB1/IGKV3D-20/IGKV1D-33/IGHV1-3/IGHV3-72/IGHV3-13/IGLV6-57/CR1/EXO1/IGLV2-11/C2/IGHV1-69D/IGLV3-25/IGHV3-73/IGHV2-5/FCER2/IGKV3-11/IGHV3-11/C3/IGLV3-21/IGKV1-39 | 6.94E-39 |
| GO:0050900 | leukocyte migration | 114 | CCL11/SELP/TREM1/ANXA1/PECAM1/DYSF/CXCL17/ADGRE2/ITGA5/SERPINE1/IL1RN/IGHV3-30/CSF3R/ITGAX/SLC7A11/CCL24/COL1A1/CXCR2/LYN/FLT1/CXCL6/S100A12/ITGA2/IGLV1-44/FPR2/SPNS2/IGHV1-46/SELE/SLC7A5/MSN/COL1A2/ANGPT2/MMP1/CD84/IGKV5-2/C5AR1/IGHV4-39/WNT5A/IGKV2-28/CCL22/PTAFR/IGLV3-1/IGHV3-48/IGLV1-40/IGKV1-12/IGHV2-70/THY1/PDE4B/S100A8/IGLV1-51/IGKV2D-28/IGKV2D-40/IGLV3-27/ICAM1/PPBP/SWAP70/IGKV2-30/SELL/S1PR1/IL23A/S100A7/MMP9/IL33/IGHV1-69/ITGB3/ITGAV/TGFB2/IGKV1-5/CCL17/CXCR5/FFAR2/IGKV1-33/LBP/IGHV4-59/TEK/IGKV2D-30/CXCR1/DOCK8/GREM1/OLR1/IGLV2-14/IGHV3-7/IGKV3-15/PLCB1/RIPOR2/LYST/NLRP12/IGKV3D-20/IGKV1D-33/CCR1/PLA2G7/ITGA4/IL1B/IGHV3-13/IGLV6-57/ITGB2/IGLV2-11/CEACAM6/NCKAP1L/S100A9/TRPV4/PIK3CG/IGLV3-25/IGHV2-5/CD300H/CCL13/IGKV3-11/CXCL9/IGHV3-11/CYP7B1/DBH/CXCL11/IGLV3-21/IGKV1-39 | 4.48E-38 |
| GO:0006959 | humoral immune response | 92 | TREM1/CD55/IGHV1-69-2/IGHV3-30/REG3A/MMP7/FCN3/C4BPA/IGHV1-24/SLC11A1/CXCL6/S100A12/AC233755.1/IGLV1-44/SPNS2/IGHV3-49/IGHV1-46/IGHV5-51/IGHG1/LYZ/IGKV5-2/C5AR1/IGHV2-26/IGHV4-39/IGKV2-28/PRSS2/IGLV3-1/IGHV3-48/IGLV1-40/DEFB4A/IGKV1-12/IGHV2-70/REG1A/S100A8/DEFA6/IGHV3-15/IGLV1-51/IGKV2D-28/CFB/IGKV2D-40/IGLV3-27/PPBP/CFI/IGKV2-30/S100A7/IGHV1-69/PGLYRP3/PTPRC/IGHV3-43/IGKV1-5/IGKV1-33/IGHV3-21/IGHV3-64/C4BPB/IGHV4-59/IGKV2D-30/IGHV3-74/IGLV2-14/IGHV1-18/IGHV3-7/IGKV3-15/HLA-DRB1/KRT6A/IGKV3D-20/IGKV1D-33/IGHV1-3/POU2F2/IGHV3-72/IGHV3-13/IGLV6-57/CR1/EXO1/IGLV2-11/PDCD1/REG1B/PAX5/C2/S100A9/IGHV1-69D/IGLV3-25/IGHV3-73/IGHV2-5/CCL13/FCER2/IGKV3-11/CXCL9/MS4A1/IGHV3-11/C3/CXCL11/IGLV3-21/IGKV1-39 | 4.52E-38 |
| GO:0006958 | complement activation, classical pathway | 59 | CD55/IGHV1-69-2/IGHV3-30/C4BPA/IGHV1-24/AC233755.1/IGLV1-44/IGHV3-49/IGHV1-46/IGHV5-51/IGHG1/IGKV5-2/IGHV2-26/IGHV4-39/IGKV2-28/IGLV3-1/IGHV3-48/IGLV1-40/IGKV1-12/IGHV2-70/IGHV3-15/IGLV1-51/IGKV2D-28/IGKV2D-40/IGLV3-27/CFI/IGKV2-30/IGHV1-69/IGHV3-43/IGKV1-5/IGKV1-33/IGHV3-21/IGHV3-64/C4BPB/IGHV4-59/IGKV2D-30/IGHV3-74/IGLV2-14/IGHV1-18/IGHV3-7/IGKV3-15/IGKV3D-20/IGKV1D-33/IGHV1-3/IGHV3-72/IGHV3-13/IGLV6-57/CR1/IGLV2-11/C2/IGHV1-69D/IGLV3-25/IGHV3-73/IGHV2-5/IGKV3-11/IGHV3-11/C3/IGLV3-21/IGKV1-39 | 2.93E-37 |
| GO:0003823 | antigen binding | 68 | IGHV1-69-2/IGHV3-30/FCN3/IGHV1-24/AC233755.1/IGLV1-44/IGHV3-49/IGHV1-46/SLC7A5/IGHV5-51/IGHG1/IGKV2-40/TAP1/IGKV5-2/IGHV2-26/IGHV4-39/IGKV2-28/IGLV3-1/CD1B/IGHV3-48/IGKV6D-21/IGLV1-40/IGKV1-12/IGHV2-70/IGHV3-15/IGLV1-51/IGKV2D-28/IGKV2D-40/IGLV3-27/HLA-DRB5/IGKV2-30/IGHV1-69/IGHV3-43/IGKV1-5/IGKV1-33/IGHV3-21/IGHV3-64/IGHV4-59/IGKV2D-30/IGHV3-74/IGKV2D-29/LILRA2/IGLV2-14/IGHV1-18/IGHV3-7/IGKV3-15/HLA-DRB1/IGKV3D-11/IGKV3D-20/IGKV1D-33/IGHV1-3/ITGA4/IGHV3-72/IGHV3-13/IGLV6-57/LILRA1/IGLV2-11/IGLV5-52/IGHV1-69D/IGLV3-25/IGHV3-73/IGHV2-5/IGKV3-11/TRGV3/IGHV3-11/LAG3/IGLV3-21/IGKV1-39 | 6.43E-35 |
| GO:0016064 | immunoglobulin mediated immune response | 71 | CD55/IGHV1-69-2/IL13RA2/IGHV3-30/C4BPA/IGHV1-24/AC233755.1/IGLV1-44/IGHV3-49/IGHV1-46/IGHV5-51/IGHG1/FOXP3/IGKV5-2/IGHV2-26/IGHV4-39/IGKV2-28/IGLV3-1/IGHV3-48/IGLV1-40/IGKV1-12/IGHV2-70/IGHV3-15/IGLV1-51/IGKV2D-28/IGKV2D-40/IGLV3-27/TFRC/SWAP70/CFI/IGKV2-30/IGHV1-69/PTPRC/IGHV3-43/IGKV1-5/IGKV1-33/IGHV3-21/IGHV3-64/C4BPB/IGHV4-59/IGKV2D-30/IGHV3-74/IGLV2-14/IGHV1-18/IGHV3-7/IGKV3-15/HLA-DRB1/IGKV3D-20/IGKV1D-33/BCL6/IGHV1-3/POU2F2/IGHV3-72/IGHV3-13/IGLV6-57/CR1/EXO1/IGLV2-11/C2/CD226/IGHV1-69D/IGLV3-25/IGHV3-73/IGHV2-5/TLR8/FCER2/IGKV3-11/IGHV3-11/C3/IGLV3-21/IGKV1-39 | 1.18E-34 |
| GO:0019724 | B cell mediated immunity | 71 | CD55/IGHV1-69-2/IL13RA2/IGHV3-30/C4BPA/IGHV1-24/AC233755.1/IGLV1-44/IGHV3-49/IGHV1-46/IGHV5-51/IGHG1/FOXP3/IGKV5-2/IGHV2-26/IGHV4-39/IGKV2-28/IGLV3-1/IGHV3-48/IGLV1-40/IGKV1-12/IGHV2-70/IGHV3-15/IGLV1-51/IGKV2D-28/IGKV2D-40/IGLV3-27/TFRC/SWAP70/CFI/IGKV2-30/IGHV1-69/PTPRC/IGHV3-43/IGKV1-5/IGKV1-33/IGHV3-21/IGHV3-64/C4BPB/IGHV4-59/IGKV2D-30/IGHV3-74/IGLV2-14/IGHV1-18/IGHV3-7/IGKV3-15/HLA-DRB1/IGKV3D-20/IGKV1D-33/BCL6/IGHV1-3/POU2F2/IGHV3-72/IGHV3-13/IGLV6-57/CR1/EXO1/IGLV2-11/C2/CD226/IGHV1-69D/IGLV3-25/IGHV3-73/IGHV2-5/TLR8/FCER2/IGKV3-11/IGHV3-11/C3/IGLV3-21/IGKV1-39 | 1.71E-34 |
| GO:0002460 | adaptive immune response based on somatic recombination of immune receptors built from immunoglobulin superfamily domains | 91 | CD55/ANXA1/IGHV1-69-2/IL13RA2/IGHV3-30/C4BPA/IGHV1-24/SLC11A1/AC233755.1/IGLV1-44/IGHV3-49/IGHV1-46/IGHV5-51/IGHG1/FOXP3/CD274/CD80/IGKV5-2/IGHV2-26/IGHV4-39/IGKV2-28/IGLV3-1/CD1B/IGHV3-48/IGLV1-40/IGKV1-12/IGHV2-70/IGHV3-15/IGLV1-51/IGKV2D-28/IGKV2D-40/IGLV3-27/TFRC/ICAM1/SWAP70/CFI/IGKV2-30/IL23A/KLHL6/IL33/IGHV1-69/PTPRC/IGHV3-43/IGKV1-5/IGKV1-33/IGHV3-21/IGHV3-64/C4BPB/IGHV4-59/IGKV2D-30/IL1RL1/IGHV3-74/UNC13D/SLAMF6/IGLV2-14/IGHV1-18/IGHV3-7/IGKV3-15/HLA-DRB1/HAVCR2/IGKV3D-20/IGKV1D-33/CLC/BCL6/IGHV1-3/POU2F2/IL1B/IGHV3-72/IGHV3-13/IGLV6-57/CR1/EXO1/ULBP3/IGLV2-11/LEF1/C2/CD226/IGHV1-69D/IGLV3-25/IRF4/IGHV3-73/RFTN1/IGHV2-5/TLR8/FCER2/IGKV3-11/IGHV3-11/C3/IGLV3-21/IGKV1-39/JAK3 | 3.76E-34 |
| GO:0050727 | regulation of inflammatory response | 98 | ADAMTS12/CD55/ANXA1/TGM2/CXCL17/SERPINE1/IGHV3-30/CDH5/CALCRL/LPL/CCL24/JAK2/C4BPA/OSMR/LYN/SUCNR1/LDLR/S100A12/ITGA2/IGLV1-44/IGHV1-46/SELE/HGF/IGHG1/IL2RA/FOXP3/BIRC3/IGKV5-2/C5AR1/IGHV4-39/WNT5A/IGKV2-28/AGT/ACE2/GBP5/IGLV3-1/TLR2/IGHV3-48/IGLV1-40/IGKV1-12/IGHV2-70/S100A8/IGLV1-51/IGKV2D-28/CFB/IGKV2D-40/IGLV3-27/MEFV/CFI/IGKV2-30/IL23A/IL33/IGHV1-69/DUOXA1/IGKV1-5/OSM/FFAR2/IGKV1-33/C4BPB/LBP/IGHV4-59/TEK/PIK3AP1/IGKV2D-30/IL1RL1/NOS2/IGLV2-14/IGHV3-7/IGKV3-15/NLRP12/IGKV3D-20/IGKV1D-33/BCL6/PLA2G7/IL1B/IGHV3-13/IGLV6-57/CR1/IGLV2-11/ADCY7/SEMA7A/IDO1/TLR6/IL21/C2/S100A9/TRPV4/ADCYAP1/PIK3CG/PTGS2/IGLV3-25/IGHV2-5/IGKV3-11/DUOXA2/IGHV3-11/C3/IGLV3-21/IGKV1-39 | 4.79E-34 |
| GO:0006956 | complement activation | 62 | CD55/IGHV1-69-2/IGHV3-30/FCN3/C4BPA/IGHV1-24/AC233755.1/IGLV1-44/IGHV3-49/IGHV1-46/IGHV5-51/IGHG1/IGKV5-2/C5AR1/IGHV2-26/IGHV4-39/IGKV2-28/IGLV3-1/IGHV3-48/IGLV1-40/IGKV1-12/IGHV2-70/IGHV3-15/IGLV1-51/IGKV2D-28/CFB/IGKV2D-40/IGLV3-27/CFI/IGKV2-30/IGHV1-69/IGHV3-43/IGKV1-5/IGKV1-33/IGHV3-21/IGHV3-64/C4BPB/IGHV4-59/IGKV2D-30/IGHV3-74/IGLV2-14/IGHV1-18/IGHV3-7/IGKV3-15/IGKV3D-20/IGKV1D-33/IGHV1-3/IGHV3-72/IGHV3-13/IGLV6-57/CR1/IGLV2-11/C2/IGHV1-69D/IGLV3-25/IGHV3-73/IGHV2-5/IGKV3-11/IGHV3-11/C3/IGLV3-21/IGKV1-39 | 8.85E-34 |
| GO:0002449 | lymphocyte mediated immunity | 89 | CD55/IGHV1-69-2/IL13RA2/IGHV3-30/SERPINB4/C4BPA/IGHV1-24/SLC11A1/AC233755.1/IGLV1-44/IGHV3-49/IGHV1-46/IGHV5-51/IGHG1/FOXP3/IGKV5-2/IGHV2-26/IGHV4-39/IGKV2-28/IGLV3-1/CD1B/IGHV3-48/IGLV1-40/IGKV1-12/IGHV2-70/IGHV3-15/IGLV1-51/IGKV2D-28/IGKV2D-40/IGLV3-27/CADM1/TFRC/ICAM1/SWAP70/CFI/IGKV2-30/IL23A/IGHV1-69/PTPRC/IGHV3-43/IGKV1-5/IGKV1-33/IGHV3-21/IGHV3-64/C4BPB/IGHV4-59/RASGRP1/IGKV2D-30/IGHV3-74/UNC13D/SLAMF6/IGLV2-14/IGHV1-18/SERPINB9/IGHV3-7/IGKV3-15/HLA-DRB1/LYST/HAVCR2/IGKV3D-20/IGKV1D-33/CLC/BCL6/IGHV1-3/POU2F2/IL1B/IGHV3-72/IGHV3-13/IGLV6-57/CR1/EXO1/ULBP3/IGLV2-11/IL21/C2/CD226/IGHV1-69D/IGLV3-25/IGHV3-73/RFTN1/IGHV2-5/TLR8/FCER2/IGKV3-11/IGHV3-11/C3/LAG3/IGLV3-21/IGKV1-39 | 1.74E-33 |
| GO:0072376 | protein activation cascade | 64 | VWF/CD55/IGHV1-69-2/IGHV3-30/FCN3/C4BPA/IGHV1-24/AC233755.1/IGLV1-44/IGHV3-49/IGHV1-46/IGHV5-51/IGHG1/IGKV5-2/C5AR1/IGHV2-26/IGHV4-39/IGKV2-28/IGLV3-1/IGHV3-48/F3/IGLV1-40/IGKV1-12/IGHV2-70/IGHV3-15/IGLV1-51/IGKV2D-28/CFB/IGKV2D-40/IGLV3-27/CFI/IGKV2-30/IGHV1-69/IGHV3-43/IGKV1-5/IGKV1-33/IGHV3-21/IGHV3-64/C4BPB/IGHV4-59/IGKV2D-30/IGHV3-74/IGLV2-14/IGHV1-18/IGHV3-7/IGKV3-15/IGKV3D-20/IGKV1D-33/IGHV1-3/IGHV3-72/IGHV3-13/IGLV6-57/CR1/IGLV2-11/C2/IGHV1-69D/IGLV3-25/IGHV3-73/IGHV2-5/IGKV3-11/IGHV3-11/C3/IGLV3-21/IGKV1-39 | 3.65E-32 |
| GO:0006909 | phagocytosis | 85 | ANXA1/IGHV1-69-2/PECAM1/DYSF/TGM2/IGHV3-30/FCN3/C4BPA/IGHV1-24/LYN/SLC11A1/ITGA2/AC233755.1/IGLV1-44/IGHV3-49/IGHV1-46/IGHV5-51/IGHG1/IGKV5-2/IGHV2-26/CLEC7A/IGHV4-39/IGKV2-28/FCGR2A/NCF2/IGLV3-1/TLR2/IGHV3-48/IGLV1-40/IGKV1-12/IGHV2-70/CD93/SYT11/IGHV3-15/IGLV1-51/IGKV2D-28/IGKV2D-40/IGLV3-27/ALOX15/FGR/IGKV2-30/IGHV1-69/PTPRC/ITGB3/ITGAV/IGHV3-43/IGKV1-5/DOCK2/IGKV1-33/IGHV3-21/IGHV3-64/C4BPB/LBP/IGHV4-59/IGKV2D-30/IGHV3-74/UNC13D/IGLV2-14/IGHV1-18/IGHV3-7/IGKV3-15/FCGR3A/IGKV3D-20/IGKV1D-33/IGHV1-3/PLCG2/IL1B/IGHV3-72/IGHV3-13/IGLV6-57/ITGB2/IGLV2-11/NCKAP1L/C2/IGHV1-69D/IGLV3-25/IGHV3-73/IGHV2-5/TXNDC5/IGKV3-11/IGHV3-11/C3/IGLV3-21/CEACAM4/IGKV1-39 | 2.58E-30 |
| GO:0009897 | external side of plasma membrane | 85 | SELP/ANXA1/IGHV1-69-2/PECAM1/ITGA5/ACKR4/IL13RA2/IGHV3-30/CSF3R/CDH5/ITGAX/HEG1/MCAM/ADAM9/CXCR2/IGHV1-24/OSMR/LDLR/SERPINE2/ITGA2/AC233755.1/IGHV3-49/IGHV1-46/IGHV5-51/IGHG1/IL2RA/CD274/CCR3/CD80/IGHV2-26/IGHV4-39/IGHV3-48/F3/IGHV2-70/THY1/CTLA4/IGHV3-15/TFRC/ICAM1/TNFRSF9/VTCN1/SELL/S1PR1/IGHV1-69/PTPRC/IL12RB2/ITGAV/IGHV3-43/CXCR5/IGHV3-21/IGHV3-64/IGHV4-59/CCR8/CXCR1/IL1RL1/IGHV3-74/IGHV1-18/IGHV3-7/HLA-DRB1/FCGR3A/CCR1/IGHV1-3/IGHV3-72/IGHV3-13/WNT2/ITGB2/CD86/SEMA7A/PDCD1/ITGB6/CD226/TMC1/ADGRE1/IGHV1-69D/IGHV3-73/IGHV2-5/TLR8/FCER2/CXCL9/MS4A1/IGHV3-11/ICOS/LAG3/PCSK9/SERPINA5 | 2.61E-29 |
| GO:0002526 | acute inflammatory response | 63 | TREM1/CD55/IL1RN/IGHV3-30/REG3A/C4BPA/CXCR2/OSMR/IGLV1-44/IGHV1-46/IGHG1/IGKV5-2/C5AR1/IGHV4-39/IGKV2-28/IGLV3-1/IGHV3-48/F3/IGLV1-40/IGKV1-12/IGHV2-70/S100A8/IGLV1-51/IGKV2D-28/CFB/IGKV2D-40/IGLV3-27/SAA4/ICAM1/CFI/IGKV2-30/IGHV1-69/IGKV1-5/OSM/FFAR2/IGKV1-33/C4BPB/LBP/IGHV4-59/IGKV2D-30/IL1A/IGLV2-14/IGHV3-7/IGKV3-15/IGKV3D-20/IGKV1D-33/IL1B/IGHV3-13/SAA2/IGLV6-57/CR1/IGLV2-11/C2/ADCYAP1/PIK3CG/PTGS2/IGLV3-25/IGHV2-5/IGKV3-11/IGHV3-11/C3/IGLV3-21/IGKV1-39 | 6.62E-28 |
| GO:0030198 | extracellular matrix organization | 83 | VWF/MMP10/SERPINB5/MMP3/PDPN/TNC/LOXL2/ERO1A/COL4A1/PXDN/PECAM1/COL4A2/COL5A3/MMP12/COL6A3/ITGA5/KDR/COL18A1/SERPINE1/OLFML2B/LAMC2/FAP/ITGAX/MMP7/ADAMTS9/SPARC/NID1/TIMP1/COL1A1/TGFBI/COL5A2/SFRP2/ITGA2/COL8A1/COL15A1/COL7A1/COL1A2/ADAMTS2/MMP1/CTSK/SH3PXD2B/FBN1/COL12A1/AGT/PRSS2/BGN/ADAMTS4/LAMA3/MMP19/LOX/ICAM1/SPP1/VCAN/MMP9/CSGALNACT1/COL4A4/ITGB3/ITGAV/TGFB2/ITGA11/ADAM19/TNFRSF11B/ADAM12/COL11A1/ACAN/GREM1/MMP13/ITGA4/ITGB2/SULF1/FBN2/LCP1/ITGB6/FSCN1/COL19A1/NPHS1/PRSS1/HSPG2/HAS1/COL4A3/ADAMTS5/NID2/EGFL6 | 7.65E-28 |
| GO:0030449 | regulation of complement activation | 45 | CD55/IGHV3-30/C4BPA/IGLV1-44/IGHV1-46/IGHG1/IGKV5-2/C5AR1/IGHV4-39/IGKV2-28/IGLV3-1/IGHV3-48/IGLV1-40/IGKV1-12/IGHV2-70/IGLV1-51/IGKV2D-28/CFB/IGKV2D-40/IGLV3-27/CFI/IGKV2-30/IGHV1-69/IGKV1-5/IGKV1-33/C4BPB/IGHV4-59/IGKV2D-30/IGLV2-14/IGHV3-7/IGKV3-15/IGKV3D-20/IGKV1D-33/IGHV3-13/IGLV6-57/CR1/IGLV2-11/C2/IGLV3-25/IGHV2-5/IGKV3-11/IGHV3-11/C3/IGLV3-21/IGKV1-39 | 1.59E-27 |
| GO:2000257 | regulation of protein activation cascade | 45 | CD55/IGHV3-30/C4BPA/IGLV1-44/IGHV1-46/IGHG1/IGKV5-2/C5AR1/IGHV4-39/IGKV2-28/IGLV3-1/IGHV3-48/IGLV1-40/IGKV1-12/IGHV2-70/IGLV1-51/IGKV2D-28/CFB/IGKV2D-40/IGLV3-27/CFI/IGKV2-30/IGHV1-69/IGKV1-5/IGKV1-33/C4BPB/IGHV4-59/IGKV2D-30/IGLV2-14/IGHV3-7/IGKV3-15/IGKV3D-20/IGKV1D-33/IGHV3-13/IGLV6-57/CR1/IGLV2-11/C2/IGLV3-25/IGHV2-5/IGKV3-11/IGHV3-11/C3/IGLV3-21/IGKV1-39 | 2.68E-27 |
| GO:0002920 | regulation of humoral immune response | 48 | CD55/IGHV3-30/C4BPA/IGLV1-44/SPNS2/IGHV1-46/IGHG1/IGKV5-2/C5AR1/IGHV4-39/IGKV2-28/IGLV3-1/IGHV3-48/IGLV1-40/IGKV1-12/IGHV2-70/IGLV1-51/IGKV2D-28/CFB/IGKV2D-40/IGLV3-27/CFI/IGKV2-30/IGHV1-69/PTPRC/IGKV1-5/IGKV1-33/C4BPB/IGHV4-59/IGKV2D-30/IGLV2-14/IGHV3-7/IGKV3-15/IGKV3D-20/IGKV1D-33/IGHV3-13/IGLV6-57/CR1/IGLV2-11/C2/IGLV3-25/IGHV2-5/FCER2/IGKV3-11/IGHV3-11/C3/IGLV3-21/IGKV1-39 | 4.29E-27 |
| GO:0002673 | regulation of acute inflammatory response | 52 | CD55/IGHV3-30/C4BPA/OSMR/IGLV1-44/IGHV1-46/IGHG1/IGKV5-2/C5AR1/IGHV4-39/IGKV2-28/IGLV3-1/IGHV3-48/IGLV1-40/IGKV1-12/IGHV2-70/IGLV1-51/IGKV2D-28/CFB/IGKV2D-40/IGLV3-27/CFI/IGKV2-30/IGHV1-69/IGKV1-5/OSM/FFAR2/IGKV1-33/C4BPB/IGHV4-59/IGKV2D-30/IGLV2-14/IGHV3-7/IGKV3-15/IGKV3D-20/IGKV1D-33/IL1B/IGHV3-13/IGLV6-57/CR1/IGLV2-11/C2/ADCYAP1/PIK3CG/PTGS2/IGLV3-25/IGHV2-5/IGKV3-11/IGHV3-11/C3/IGLV3-21/IGKV1-39 | 5.46E-27 |
| GO:0043062 | extracellular structure organization | 87 | VWF/MMP10/SERPINB5/MMP3/LIPG/PDPN/TNC/LOXL2/ERO1A/COL4A1/PXDN/PECAM1/COL4A2/COL5A3/MMP12/COL6A3/ITGA5/KDR/COL18A1/SERPINE1/OLFML2B/LAMC2/FAP/ITGAX/MMP7/ADAMTS9/LPL/SPARC/NID1/TIMP1/COL1A1/TGFBI/COL5A2/SFRP2/ITGA2/COL8A1/COL15A1/COL7A1/COL1A2/ADAMTS2/MMP1/CTSK/SH3PXD2B/FBN1/COL12A1/AGT/PRSS2/BGN/ADAMTS4/LAMA3/MMP19/LOX/ICAM1/SPP1/VCAN/MMP9/CSGALNACT1/COL4A4/ITGB3/ITGAV/TGFB2/ITGA11/ADAM19/TNFRSF11B/ADAM12/COL11A1/ACAN/GREM1/MMP13/PLA2G7/ITGA4/HYAL1/ITGB2/SULF1/FBN2/LCP1/ITGB6/FSCN1/COL19A1/NPHS1/PRSS1/HSPG2/HAS1/COL4A3/ADAMTS5/NID2/EGFL6 | 3.44E-26 |
| GO:0002697 | regulation of immune effector process | 91 | CD55/ANXA1/MMP12/ADGRE2/IL13RA2/IGHV3-30/SERPINB4/C4BPA/LYN/IRAK3/CXCL6/IGLV1-44/IGHV1-46/IGHG1/IL2RA/FOXP3/BIRC3/CD84/CD80/IGKV5-2/C5AR1/IGHV4-39/WNT5A/IGKV2-28/STAT1/PTAFR/IGLV3-1/CD1B/IGHV3-48/IGLV1-40/IGKV1-12/IGHV2-70/IGLV1-51/IGKV2D-28/CFB/IGKV2D-40/IGLV3-27/CADM1/TFRC/FGR/CFI/IGKV2-30/IL23A/IL33/IGHV1-69/PGLYRP3/PTPRC/TGFB2/IGKV1-5/FFAR2/IGKV1-33/C4BPB/LBP/IGHV4-59/RASGRP1/IGKV2D-30/UNC13D/SLAMF6/IGLV2-14/SERPINB9/IGHV3-7/IGKV3-15/HAVCR2/IGKV3D-20/IGKV1D-33/CLC/BCL6/IL1B/IGHV3-13/IGLV6-57/CR1/ITGB2/CD86/ULBP3/IGLV2-11/SEMA7A/IL21/C2/CD226/IGLV3-25/IRF4/IGHV2-5/FCER2/IGKV3-11/IGHV3-11/C3/LAG3/IGLV3-21/IGKV1-39/JAK3/AIM2 | 6.03E-26 |
| GO:0004252 | serine-type endopeptidase activity | 65 | MMP10/MMP3/MMP12/IGHV3-30/FAP/MMP7/FCN3/PLAU/IGLV1-44/IGHV1-46/HGF/HTRA3/IGHG1/MMP1/CTSK/IGKV5-2/IGHV4-39/IGKV2-28/PRSS2/PCSK1/IGLV3-1/IGHV3-48/F3/IGLV1-40/TMPRSS3/IGKV1-12/IGHV2-70/MMP19/IGLV1-51/IGKV2D-28/CFB/IGKV2D-40/IGLV3-27/CFI/IGKV2-30/MMP9/IGHV1-69/IGKV1-5/IGKV1-33/IGHV4-59/KLK12/IGKV2D-30/IGLV2-14/KLK11/KLK10/IGHV3-7/IGKV3-15/MMP13/IGKV3D-20/IGKV1D-33/IGHV3-13/IGLV6-57/IGLV2-11/C2/IGLV3-25/IGHV2-5/PRSS1/IGKV3-11/IGHV3-11/C3/PCSK9/IGLV3-21/GZMH/GZMK/IGKV1-39 | 3.88E-26 |
| GO:0002429 | immune response-activating cell surface receptor signaling pathway | 86 | IGHV1-69-2/IGHV3-30/MUC5AC/IGHV1-24/LYN/AC233755.1/IGLV1-44/FPR2/IGHV3-49/IGHV1-46/IGHV5-51/IGHG1/FOXP3/IGKV5-2/C5AR1/IGHV2-26/IGHV4-39/IGKV2-28/FCGR2A/IGLV3-1/IGHV3-48/IGLV1-40/IGKV1-12/IGHV2-70/THY1/CTLA4/PDE4B/IGHV3-15/IGLV1-51/IGKV2D-28/IGKV2D-40/IGLV3-27/HLA-DRB5/FGR/VTCN1/IGKV2-30/KLHL6/IGHV1-69/PTPRC/IGHV3-43/IGKV1-5/FFAR2/IGKV1-33/IGHV3-21/GBP1/IGHV3-64/IGHV4-59/MUC5B/IGKV2D-30/IGHV3-74/ITK/LILRA2/CLEC4D/IGLV2-14/IGHV1-18/FPR1/IGHV3-7/CLEC6A/IGKV3-15/HLA-DRB1/MUC16/FCGR3A/IGKV3D-20/IGKV1D-33/IGHV1-3/PLCG2/CD38/IGHV3-72/IGHV3-13/IGLV6-57/CR1/IGLV2-11/PAX5/NCKAP1L/CD226/IGHV1-69D/IGLV3-25/IGHV3-73/RFTN1/IGHV2-5/IGKV3-11/IGHV3-11/CARD11/IGLV3-21/PRKCB/IGKV1-39 | 4.11E-25 |
| GO:0070613 | regulation of protein processing | 53 | CD55/SERPINE1/IGHV3-30/C4BPA/SERPINE2/IGLV1-44/IGHV1-46/RPS6KA2/IGHG1/BIRC3/IGKV5-2/C5AR1/IGHV4-39/IGKV2-28/IGLV3-1/IGHV3-48/IGLV1-40/IGKV1-12/IGHV2-70/IGLV1-51/IGKV2D-28/CFB/IGKV2D-40/IGLV3-27/CFI/IGKV2-30/CHAC1/IGHV1-69/LDLRAD3/IGKV1-5/IGKV1-33/C4BPB/IGHV4-59/IGKV2D-30/IGLV2-14/IGHV3-7/IGKV3-15/LRRK2/IGKV3D-20/IGKV1D-33/IGHV3-13/IGLV6-57/CR1/IGLV2-11/C2/IGLV3-25/IGHV2-5/GAS1/IGKV3-11/IGHV3-11/C3/IGLV3-21/IGKV1-39 | 7.34E-25 |
| GO:0002768 | immune response-regulating cell surface receptor signaling pathway | 89 | IGHV1-69-2/IGHV3-30/MUC5AC/LILRB2/IGHV1-24/LYN/AC233755.1/IGLV1-44/FPR2/IGHV3-49/IGHV1-46/IGHV5-51/IGHG1/FOXP3/IGKV5-2/C5AR1/IGHV2-26/IGHV4-39/IGKV2-28/FCGR2A/IGLV3-1/IGHV3-48/IGLV1-40/IGKV1-12/IGHV2-70/THY1/CTLA4/PDE4B/IGHV3-15/IGLV1-51/IGKV2D-28/IGKV2D-40/IGLV3-27/HLA-DRB5/FGR/VTCN1/IGKV2-30/KLHL6/IGHV1-69/PTPRC/IGHV3-43/IGKV1-5/FFAR2/IGKV1-33/IGHV3-21/GBP1/IGHV3-64/IGHV4-59/MUC5B/IGKV2D-30/IGHV3-74/ITK/LILRA2/CLEC4D/IGLV2-14/IGHV1-18/FPR1/IGHV3-7/CLEC6A/IGKV3-15/HLA-DRB1/MUC16/FCGR3A/IGKV3D-20/IGKV1D-33/IGHV1-3/PLCG2/CD38/IGHV3-72/IGHV3-13/IGLV6-57/CR1/IGLV2-11/NFATC1/PAX5/NCKAP1L/CD226/IGHV1-69D/IGLV3-25/IGHV3-73/RFTN1/IGHV2-5/IGKV3-11/NFATC3/IGHV3-11/CARD11/IGLV3-21/PRKCB/IGKV1-39 | 1.37E-24 |
| GO:1903317 | regulation of protein maturation | 53 | CD55/SERPINE1/IGHV3-30/C4BPA/SERPINE2/IGLV1-44/IGHV1-46/RPS6KA2/IGHG1/BIRC3/IGKV5-2/C5AR1/IGHV4-39/IGKV2-28/IGLV3-1/IGHV3-48/IGLV1-40/IGKV1-12/IGHV2-70/IGLV1-51/IGKV2D-28/CFB/IGKV2D-40/IGLV3-27/CFI/IGKV2-30/CHAC1/IGHV1-69/LDLRAD3/IGKV1-5/IGKV1-33/C4BPB/IGHV4-59/IGKV2D-30/IGLV2-14/IGHV3-7/IGKV3-15/LRRK2/IGKV3D-20/IGKV1D-33/IGHV3-13/IGLV6-57/CR1/IGLV2-11/C2/IGLV3-25/IGHV2-5/GAS1/IGKV3-11/IGHV3-11/C3/IGLV3-21/IGKV1-39 | 1.45E-24 |
| GO:0008236 | serine-type peptidase activity | 65 | MMP10/MMP3/MMP12/IGHV3-30/FAP/MMP7/FCN3/PLAU/IGLV1-44/IGHV1-46/HGF/HTRA3/IGHG1/MMP1/CTSK/IGKV5-2/IGHV4-39/IGKV2-28/PRSS2/PCSK1/IGLV3-1/IGHV3-48/F3/IGLV1-40/TMPRSS3/IGKV1-12/IGHV2-70/MMP19/IGLV1-51/IGKV2D-28/CFB/IGKV2D-40/IGLV3-27/CFI/IGKV2-30/MMP9/IGHV1-69/IGKV1-5/IGKV1-33/IGHV4-59/KLK12/IGKV2D-30/IGLV2-14/KLK11/KLK10/IGHV3-7/IGKV3-15/MMP13/IGKV3D-20/IGKV1D-33/IGHV3-13/IGLV6-57/IGLV2-11/C2/IGLV3-25/IGHV2-5/PRSS1/IGKV3-11/IGHV3-11/C3/PCSK9/IGLV3-21/GZMH/GZMK/IGKV1-39 | 1.08E-23 |
| GO:0017171 | serine hydrolase activity | 65 | MMP10/MMP3/MMP12/IGHV3-30/FAP/MMP7/FCN3/PLAU/IGLV1-44/IGHV1-46/HGF/HTRA3/IGHG1/MMP1/CTSK/IGKV5-2/IGHV4-39/IGKV2-28/PRSS2/PCSK1/IGLV3-1/IGHV3-48/F3/IGLV1-40/TMPRSS3/IGKV1-12/IGHV2-70/MMP19/IGLV1-51/IGKV2D-28/CFB/IGKV2D-40/IGLV3-27/CFI/IGKV2-30/MMP9/IGHV1-69/IGKV1-5/IGKV1-33/IGHV4-59/KLK12/IGKV2D-30/IGLV2-14/KLK11/KLK10/IGHV3-7/IGKV3-15/MMP13/IGKV3D-20/IGKV1D-33/IGHV3-13/IGLV6-57/IGLV2-11/C2/IGLV3-25/IGHV2-5/PRSS1/IGKV3-11/IGHV3-11/C3/PCSK9/IGLV3-21/GZMH/GZMK/IGKV1-39 | 3.50E-23 |
| GO:0098542 | defense response to other organism | 88 | SELP/IGHV1-69-2/MMP12/DDIT4/OAS3/SERPINE1/IGHV3-30/ITGAX/KCNJ8/MMP7/FCN3/IGHV1-24/SLC11A1/CXCL6/S100A12/AC233755.1/IGHV3-49/IGHV1-46/IGHV5-51/IGHG1/IL2RA/BNIP3/LYZ/BIRC3/C5AR1/IGHV2-26/CLEC7A/IGHV4-39/STAT1/TLR2/APOBEC1/IGHV3-48/DEFB4A/IGHV2-70/S100A8/SYT11/DEFA6/OAS2/TNFSF8/IGHV3-15/SLFN11/FGR/PPBP/ARG2/ABCC9/IL23A/S100A7/PMAIP1/IL33/IGHV1-69/PGLYRP3/PTPRC/IGHV3-43/IGHV3-21/GBP1/IGHV3-64/LBP/IGHV4-59/EPHA2/IGHV3-74/NOS2/UNC13D/CLEC4D/IGHV1-18/IGHV3-7/CLEC6A/LYST/KRT6A/HAVCR2/IGHV1-3/IL1B/IGHV3-72/IGHV3-13/OASL/TLR6/S100A9/IGHV1-69D/APOBEC3A/IRF4/IGHV3-73/IGHV2-5/TLR8/VGF/CXCL9/IGHV3-11/ADAMTS5/AIM2/IFIT3 | 3.21E-21 |
