# Supplementary material for: Transcriptomic analyses of treatment-naïve pediatric ulcerative colitis patients and exploration of underlying disease pathogenesis
Source: J Transl Med. 2023 Jan 16;21:30. doi: 10.1186/s12967-023-03881-6 (PMC9843999; doi:10.1186/s12967-023-03881-6)
Supplement: Supplementary file 5 — Additional file 5: Table S5. KEGG pathway enrichment of upregulated mRNA in UC group and control group. [file 12967_2023_3881_MOESM5_ESM.doc]

| **Table S5.** KEGG pathway enrichment of upregulated mRNA in UC group and control group | | | |
| --- | --- | --- | --- |
| Pathway ID | Pathway | GeneRatio | padj |
| hsa05150 | Staphylococcus aureus infection | 16/486 | 4.15E-07 |
| hsa05140 | Leishmaniasis | 20/486 | 4.15E-07 |
| hsa04060 | Cytokine-cytokine receptor interaction | 46/486 | 5.94E-07 |
| hsa04514 | Cell adhesion molecules (CAMs) | 29/486 | 5.94E-07 |
| hsa04512 | ECM-receptor interaction | 22/486 | 2.37E-06 |
| hsa05323 | Rheumatoid arthritis | 20/486 | 3.95E-06 |
| hsa04610 | Complement and coagulation cascades | 20/486 | 4.42E-06 |
| hsa04933 | AGE-RAGE signaling pathway in diabetic complications | 23/486 | 1.22E-05 |
| hsa05146 | Amoebiasis | 22/486 | 2.00E-05 |
| hsa04640 | Hematopoietic cell lineage | 19/486 | 0.000139965 |
| hsa04151 | PI3K-Akt signaling pathway | 47/486 | 0.000186751 |
| hsa04670 | Leukocyte transendothelial migration | 20/486 | 0.002081078 |
| hsa05152 | Tuberculosis | 25/486 | 0.002102003 |
| hsa04145 | Phagosome | 22/486 | 0.002146225 |
| hsa04062 | Chemokine signaling pathway | 27/486 | 0.0038403 |
| hsa05321 | Inflammatory bowel disease (IBD) | 12/486 | 0.004442657 |
| hsa04974 | Protein digestion and absorption | 16/486 | 0.004783412 |
| hsa05144 | Malaria | 11/486 | 0.006004576 |
| hsa04510 | Focal adhesion | 28/486 | 0.00800517 |
| hsa04657 | IL-17 signaling pathway | 16/486 | 0.012183312 |
| hsa04611 | Platelet activation | 19/486 | 0.016087843 |
| hsa04066 | HIF-1 signaling pathway | 16/486 | 0.020289544 |
| hsa04659 | Th17 cell differentiation | 15/486 | 0.03720159 |
| hsa05332 | Graft-versus-host disease | 5/486 | 0.04470826 |
| hsa05414 | Dilated cardiomyopathy (DCM) | 14/486 | 0.046879692 |
