# Supplementary material for: Transcriptomic analyses of treatment-naïve pediatric ulcerative colitis patients and exploration of underlying disease pathogenesis
Source: J Transl Med. 2023 Jan 16;21:30. doi: 10.1186/s12967-023-03881-6 (PMC9843999; doi:10.1186/s12967-023-03881-6)
Supplement: Supplementary file 6 — Additional file 6: Table S6. KEGG pathway enrichment. [file 12967_2023_3881_MOESM6_ESM.doc]

| **Table S6.** KEGG pathway enrichment. | | | | |
| --- | --- | --- | --- | --- |
| Term | ID | p value | geneName | KEGG_ID |
| ECM-receptor interaction | hsa04512 | 4.17522069060846E-08 | VWF/TNC/COL4A1/COL4A2/COL6A3/ITGA5/LAMC2/COL1A1/ITGA2/COL1A2/COL6A5/LAMA3/SPP1/COL4A4/ITGB3/THBS2/ITGAV/ITGA11/ITGA4/ITGB6/HSPG2/COL4A3 | hsa:7450/hsa:3371/hsa:1282/hsa:1284/hsa:1293/hsa:3678/hsa:3918/hsa:1277/hsa:3673/hsa:1278/hsa:256076/hsa:3909/hsa:6696/hsa:1286/hsa:3690/hsa:7058/hsa:3685/hsa:22801/hsa:3676/hsa:3694/hsa:3339/hsa:1285 |
| Inflammatory bowel disease (IBD) | hsa05321 | 0.000250290553712622 | FOXP3/STAT1/TLR2/HLA-DRB5/IL23A/IL12RB2/TGFB2/IL1A/IL21R/IL1B/NFATC1/IL21 | hsa:50943/hsa:6772/hsa:7097/hsa:3127/hsa:51561/hsa:3595/hsa:7042/hsa:3552/hsa:50615/hsa:3553/hsa:4772/hsa:59067 |
