# Supplementary material for: Transcriptomic analyses of treatment-naïve pediatric ulcerative colitis patients and exploration of underlying disease pathogenesis
Source: J Transl Med. 2023 Jan 16;21:30. doi: 10.1186/s12967-023-03881-6 (PMC9843999; doi:10.1186/s12967-023-03881-6)
Supplement: Supplementary file 7 — Additional file 7: Table S7. Clinical characteristics of the patients involved in sequencing. [file 12967_2023_3881_MOESM7_ESM.doc]

| **Table S7. Clinical characteristics of the patients involved in sequencing.** | |  |
| --- | --- | --- |
|  | Control | UC |
|  | (n = 5) | (n = 5) |
| Gender (female/male) | 2/3 | 2/3 |
| Median age (range), years | 12.1 (7.92 -14.75) | 13.1 (8.0 - 14.75) |
| Paediatric Ulcerative Colitis Activity Index (PUCAI) | - | 30(20-60) |
| Mayo score | - | 2(1-2) |
| Median ESR (range), mm/h | - | 32(9.0-120.0) |
| Median CRP (range), mg/L | - | 3.02 (3.02 - 39.49) |
| Peripheral blood cell count |  |  |
| White blood cell count (×10^12 cells/L) | 6.64± 1.03 | 9.79 ± 5.54 |
| Blood neutrophil count (%) | 50(43-59) | 54 (45 - 84) |
| Blood lymphocyte count (%) | 34(31-47) | 26 (10-47) |
| Blood eosinophil count (%) | 2.7(1-8.5) | 3.9 (0 - 11.1) |
| Red blood cell count (×10^9 cells/L) | 4.79 ± 0.68 | 4.41 ± 0.05 |
| Hemoglobin (g/L) | 137.6 ± 19.7 | 107.2 ± 24.0 |
| Blood platelet count (×10^9 cells/L) | 308.2 ± 33.0 | 452.0 ± 154.9 |
| Quantitative data with a normal distribution are presented as mean ± SD. Quantitative data with a non-normal distribution are presented as median. | | |
