# Supplementary material for: Transcriptomic analyses of treatment-naïve pediatric ulcerative colitis patients and exploration of underlying disease pathogenesis
Source: J Transl Med. 2023 Jan 16;21:30. doi: 10.1186/s12967-023-03881-6 (PMC9843999; doi:10.1186/s12967-023-03881-6)
Supplement: Supplementary file 8 — Additional file 8: Table S8. Primers used in qRT-PCR. [file 12967_2023_3881_MOESM8_ESM.doc]

**Table S8. Primers used in qRT-PCR**

|  | Gene name |  | Sequence (5' -> 3') | Amplicon Size |
| --- | --- | --- | --- | --- |
| 1 | PIK3CD | Forward Primer | GCTTCTCTTCCTCCACCTCTTTGC | 131 |
|  |  | Reverse Primer | AGTGCTTCAGTGCCTCCTCCTC |  |
| 2 | IL1α | Forward Primer | GACTGCCCAAGATGAAGACCAACC | 109 |
|  |  | Reverse Primer | GCCGTGAGTTTCCCAGAAGAAGAG |  |
| 3 | IL1β | Forward Primer | CTCCACCTCCAGGGACAGGATATG | 138 |
|  |  | Reverse Primer | TTTCAACACGCAGGACAGGTACAG |  |
| 4 | TIMP1 | Forward Primer | ACACTGTTGGCTGTGAGGAATGC | 138 |
|  |  | Reverse Primer | AGTTCCTGGTCCTCATCTCGTCTG |  |
| 5 | COL6A3 | Forward Primer | AGCTCCTCCTGGTCTGCGTTC | 121 |
|  |  | Reverse Primer | TGCCTCTGGGTCCTGGTTTTCC |  |
| 6 | MMP1 | Forward Primer | ACCATGCCATTGAGAAAGCCTTCC | 80 |
|  |  | Reverse Primer | TGCTTGACCCTCAGAGACCTTGG |  |
| 7 | MMP12 | Forward Primer | TTTTGGACCTGGATCTGGCATTGG | 105 |
|  |  | Reverse Primer | TCGTGAACAGCAGTGAGGAACAAG |  |
| 8 | HLA-DRB5 | Forward Primer | CAGTGCAGCGGCGAGTTGAG | 95 |
|  |  | Reverse Primer | ACCATTCACAGAGCAGACCAGGAG |  |
